# T4SS-dependent TLR5 activation by *Helicobacter pylori* infection

**DOI:** 10.1038/s41467-019-13506-6

**Published:** 2019-12-16

**Authors:** Suneesh Kumar Pachathundikandi, Nicole Tegtmeyer, Isabelle Catherine Arnold, Judith Lind, Matthias Neddermann, Christina Falkeis-Veits, Sujay Chattopadhyay, Mark Brönstrup, Werner Tegge, Minsun Hong, Heinrich Sticht, Michael Vieth, Anne Müller, Steffen Backert

**Affiliations:** 1 Department of Biology, Division of Microbiology, Friedrich Alexander University Erlangen-Nuremberg, Erlangen, Germany; 2 Institute of Molecular Cancer Research, University of Zurich, Zurich, Switzerland; 3 Institute for Pathology, Klinikum Bayreuth, Bayreuth, Germany; 4 JIS Institute of Advanced Studies and Research, JIS University, Kolkata, 700091 India; 5 Department of Chemical Biology, Helmholtz Centre for Infection Research, Braunschweig, Germany; 6 Division of Biological Science and Technology, Yonsei University, Wonju, Republic of Korea; 7 Institute of Biochemistry, Division of Bioinformatics, Friedrich Alexander University Erlangen-Nuremberg, Erlangen, Germany

**Keywords:** Toll-like receptors, Pathogens

## Abstract

Toll-like receptor TLR5 recognizes a conserved domain, termed D1, that is present in flagellins of several pathogenic bacteria but not in *Helicobacter pylori*. Highly virulent *H. pylori* strains possess a type IV secretion system (T4SS) for delivery of virulence factors into gastric epithelial cells. Here, we show that one of the *H. pylori* T4SS components, protein CagL, can act as a flagellin-independent TLR5 activator. CagL contains a D1-like motif that mediates adherence to TLR5^+^ epithelial cells, TLR5 activation, and downstream signaling in vitro. TLR5 expression is associated with *H. pylori* infection and gastric lesions in human biopsies. Using *Tlr5*-knockout and wild-type mice, we show that TLR5 is important for efficient control of *H. pylori* infection. Our results indicate that CagL, by activating TLR5, may modulate immune responses to *H. pylori*.

## Abstract (web summary)

Toll-like receptor TLR5 recognizes a domain, D1, that is present in flagellins of several pathogenic bacteria but not in *Helicobacter pylori*. Here, the authors show that TLR5 can be activated independently of flagellin by a component of the *H. pylori* type IV secretion system that contains a D1-like motif.

## Introduction

*Helicobacter pylori* is a paradigm persistent pathogen colonizing about 50% of the human world population and represents a major risk factor for chronic gastritis, peptic ulceration and gastric malignancies, but host factors controlling the infection and disease development remain obscure^1,2^. Toll-like receptors (TLRs) are innate immune receptors for the detection of invading microbes^3–5^. TLR5 specifically detects a conserved motif present in bacterial flagellins, termed αD1a, that is expressed by *Salmonella, Vibrio* and other human-pathogenic species. In contrast, *H. pylori* has evolutionarily acquired specific mutations in the D1 interaction domain of its flagellin FlaA to evade immune detection by TLR5 without compromising flagellar motility^6,7^. Highly virulent *H. pylori* strains harbor the cytotoxin-associated gene pathogenicity island (*cag*PAI), which encodes a type IV secretion system (T4SS), forming a syringe-like pilus structure for translocation of virulence factors such as CagA and LPS metabolites into gastric epithelial cells^8–10^. The T4SS pilus-exposed protein CagL is required to establish host cell contact and drives effector delivery^11^. A role of TLR5 during infection with *H. pylori* has been repeatedly excluded due to the low intrinsic activity of its flagellin^6,7,12–14^. However, we have reported previously that *H. pylori* infection upregulates the expression of TLR5 both on epithelial and immune cells^15^. This raised the question which bacterial factor activates TLR5.

The T4SS delivers multiple factors to the host cytoplasm, which ensues different cellular signaling for disease formation and progression^8–10^. Here we show that the T4SS pilus tip protein CagL is necessary for NF-κB activation in a TLR5-dependent manner. CagL sequences from various *H. pylori* strains around the globe harbor a D1-like motif, which we show is required for TLR5 signaling and NF-κB activation. In addition, assays using human gastric biopsies and mouse models indicate that TLR5 activation and signaling may be important for *H. pylori* infection and the associated immune responses.

## Results and Discussion

### *H. pylori* activates NF-κB in CagL-dependent TLR5 signaling

**Fig. 1:**
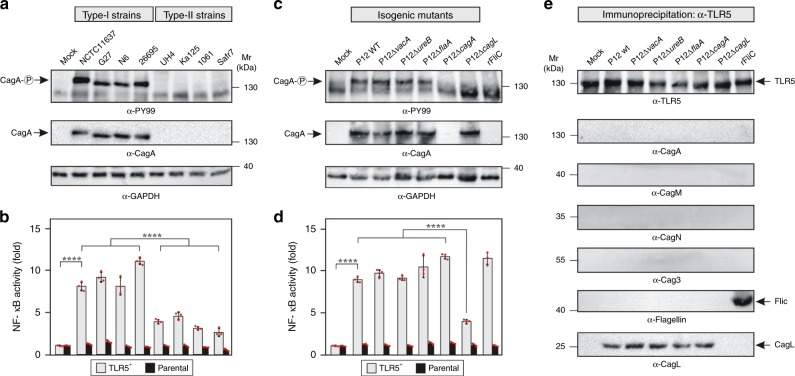
Activation of TLR5 by *cag*PAI-positive, but not *cag*PAI-negative *H. pylori* strains. **a** *H. pylori* type-I, but not type-II strains, express a functional T4SS allowing for CagA phosphorylation in infected AGS cells as control. **b** TLR5 activation as quantified by NF-κB luciferase reporter gene assay is restricted to type-I isolates in HEK reporter cells. **c** CagA delivery and its phosphorylation in AGS cells require functional *cagL* and *cagA*, but not *vacA*, *ureB* and *flaA* genes. **d** TLR5 activation in HEK293 cells requires functional *cagL*. Recombinant flagellin (rFliC) of *Salmonella* was used as positive control. **e**, CagL and rFliC interact with TLR5 on TLR5^+^ cells as determined by immunoprecipitation with α-TLR5 antibodies. Quantitative data are shown as means ± SD. ****p < 0.0001 (one-way ANOVA). Each red dot represents a single data point. Source data are provided as a Source Data file.

To examine which bacterial factor in *H. pylori* activates TLR5, we utilized established HEK293 reporter cells stably transfected with TLR5 (TLR5^+^) and additionally expressing a luciferase reporter for the pro-inflammatory transcription factor NF-κB^7,12–15^. This system has also the advantage that CagA and LPS metabolites cannot be translocated into HEK293 cells^15,16^. Thus, monitoring of TLR5 activation in these cells is ideally not affected by other T4SS functions. TLR5 activation was continuously increasing over 8 h and depended on the multiplicity of infection (Supplementary Fig. 1). Whereas highly virulent *cag*PAI-positive clinical isolates specifically activated NF-κB in TLR5^+^ cells compared to the parental controls, isolates lacking the *cag*PAI showed only background NF-κB activation (Fig. 1a, b). Any delay in TLR5 activation upon infection with various strains was not seen. These findings suggest that the T4SS is required for TLR5 activation. Next, we infected TLR5^+^ and parental cells with wild-type (WT) *H. pylori* and isogenic mutants of well-known virulence genes. Whereas the inactivation of *vacA*, *ureB, flaA* and *cagA* did not abolish TLR5 activation, the loss of *cagL* and of various structural T4SS genes abrogated TLR5 activation (Fig. 1c, d and Supplementary Figs. 2 and 3). Remarkably, activation of TLR5 by the *cag*PAI was as strong as its activation by recombinant *Salmonella* flagellin (rFliC) (Fig. 1d, Supplementary Fig. 4). As another control, infection of HEK293-TLR4^+^ reporter cells revealed T4SS-independent NF-κB activation and no activation by FliC (Supplementary Fig. 5). To identify the bacterial binding partner of TLR5, cells were infected or treated with rFliC and subjected to immunoprecipitation using TLR5-specific antibodies. Equal amounts of TLR5 were precipitated (Fig. 1e). Re-probing of the blot revealed a robust signal for CagL, but not other *cag*PAI proteins (Fig. 1e). As expected, rFliC was also co-precipitated, while FlaA^*Hp*^ did not (Fig. 1e). This suggests that CagL physically interacts with TLR5 on intact infected epithelial cells.

### Identification of TLR5 binding D1-motif in *H. pylori* CagL

**Fig. 2:**
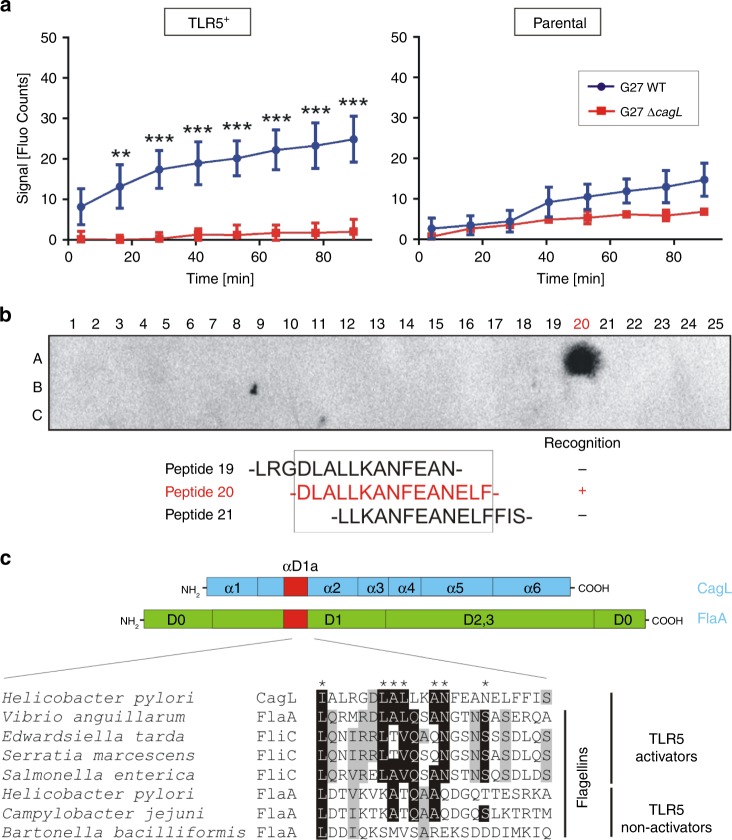
The T4SS-pilus protein CagL of *H. pylori* interacts directly with TLR5. **a** Binding of FITC-labeled *H. pylori* G27 WT or Δ*cagL* mutant to parental and TLR5^+^ cells, as monitored over time using the LigandTracer system under live cell conditions. **b** Peptide arrays of overlapping CagL 15-mer peptides were incubated with recombinant His-tagged TLR5. Probing with α-His antibodies revealed binding of TLR5 to peptide-20, but not the partially overlapping peptides-19 or −21. **c** The peptide-20 sequence of CagL shows homology to the flagellin αD1a domains of TLR5-activating, but not TLR5-non-activating flagellins. Quantitative data are shown as means ± SD. **p < 0.01; ***p < 0.001 (two-way ANOVA). Source data are provided as a Source Data file.

We next monitored the interaction of fluorescence-labeled bacteria with TLR5 using live cell imaging^17^. Infection of parental cells revealed low fluorescence signals and low binding affinity of both WT *H. pylori* and Δ*cagL* mutant. In contrast, WT but not Δ*cagL* mutant bacteria attached firmly to TLR5^+^ cells (Fig. 2a). These data demonstrate that expression of TLR5 permits efficient attachment of *H. pylori* to TLR5^+^ cells, with CagL serving as the TLR5 ligand on the bacterial surface. This was confirmed by cell binding assays^18^ in a time course, showing that TLR5 expression was required for potent attachment of WT compared to Δ*cagL* mutant (Supplementary Fig. 6). To investigate which CagL-motifs interact with TLR5, we performed peptide arrays^19^. To this end, overlapping 15-mer peptides covering the CagL sequence were spotted on a membrane and probed with recombinant TLR5 (rTLR5) (Supplementary Figs. 7 and 8). Interestingly, rTLR5 bound strongly to the array peptide no. 20 (Fig. 2b). A database search of the peptide’s sequence (DLALLKANFEANELF) revealed a remarkable conservation across CagL from isolates collected in various geographical areas of the world (Supplementary Fig. 9) and high similarity to αD1a domains of TLR5-activating flagellins from *Vibrio*, *Edwardsiella*, *Serratia* and *Salmonella* species (in particular an 8-residue “DLALLKAN” sequence, representing a TLR5-activating motif). In contrast, this motif is absent from FlaA^*Hp*^ and other TLR5-non-activating flagellins of *Campylobacter jejuni* and *Bartonella bacilliformis* (Fig. 2c). Structural inspection of a complex crystal structure between *Salmonella* FliC and TLR5 indicates that the respective flagellin sequence stretch directly interacts with TLR5 (Supplementary Fig. 10a, b). As noted previously^13^, this stretch contains one leucine residue, which is highly conserved in various TLR5-activating flagellins and in CagL (L79), but is absent in the non-activating flagellins (Fig. 2c). This leucine forms hydrophobic interactions with F278 of TLR5 (Supplementary Fig. 10c), whereas the presence of a lysine (K95), as observed in FlaA^*Hp*^, would place a positive charge in the proximity of the hydrophobic F278 sidechain (Supplementary Fig. 10d), thus resulting in an unfavorable interaction and abrogated TLR5 activation. These results suggest that *H. pylori* avoids detection of its FlaA by TLR5, but instead expresses the T4SS protein CagL to promote binding and activation of TLR5.

### FlaA^*Hp*^ swapped with CagL D1-motif regained TLR5 activation

**Fig. 3:**
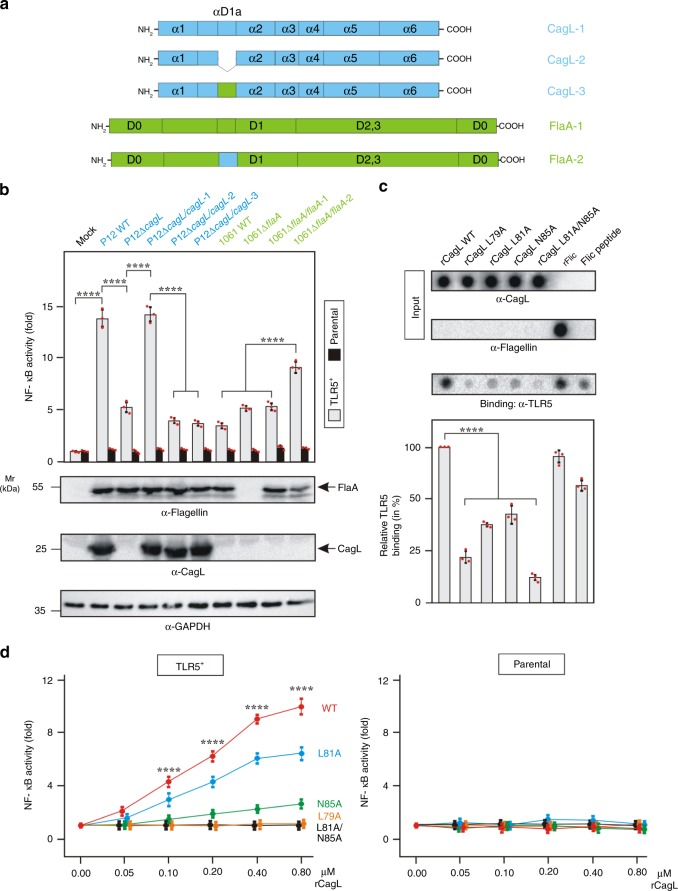
*H. pylori* CagL contains a flagellin-mimetic motif that interacts with TLR5. **a** Schematic representation of CagL and FlaA^*Hp*^ produced by deletion or swapping of the αD1a motif. **b** TLR5^+^ HEK293 cells were infected with the indicated *H. pylori* strains expressing the constructs shown in A, followed by quantification of NF-κB activity. **c** Protein binding assay. The indicated recombinant CagL proteins, FliC protein and FliC peptide as control were immobilized on Dotblots, followed by incubation with recombinant TLR5. Site-directed mutagenesis in the D1-motif of CagL reveals its importance in TLR5 binding as quantified densitometrically. **d** Dose-response curves of NF-κB activity in TLR5^+^ and parental cells treated with the indicated amounts of CagL proteins. EC_50_ value of WT CagL was determined to be 172.6 nM by using AAT Bioquest (https://www.aatbio.com). Mutations in the CagL D1-motif confirm its function in TLR5 activation. The CagL WT values are significant over all mutants as indicated. Quantitative data are shown as means ± SD. ****p < 0.0001 (one-way ANOVA). Each red dot represents a single data point. Source data are provided as a Source Data file.

To verify these findings and confirm a key role of CagL in TLR5 recognition, we swapped the proposed TLR5-activating motif (DLALLKAN) in CagL with the corresponding sequence of FlaA^*Hp*^ (VKATQAAQ, a non-TLR5-activating motif) (Fig. 3a) in the *cag*PAI-positive strain P12 or *cag*PAI-negative strain 1061, respectively. Infection of HEK293 cells showed that complementation of Δ*cagL* mutant with WT CagL restored TLR5 activation, whereas this was not the case by CagL with deletion of the DLALLKAN-motif or CagL in which the original sequence has been replaced by the FlaA^*Hp*^ sequence VKATQAAQ (Fig. 3b). Genetic complementation of WT FlaA^*Hp*^ did not activate TLR5 as expected, but introduction of the DLALLKAN sequence of CagL in FlaA^*Hp*^ restored its potential to activate TLR5 (Fig. 3b). In addition, we generated point mutations in the evolutionarily conserved residues of the CagL DLALLKAN-motif including L79A, L81A or N85A (Supplementary Fig. 11). These proteins were subjected to binding assays using Dotblots. The results show that WT CagL and rFliC as well as the corresponding D1-motif containing FliC peptide (ELAVQSANSTNSQSD) bound rTLR5 with high efficiency (Fig. 3c). Single L79 mutation in CagL had the strongest effect on rTLR5 binding, while L81A or N85A revealed intermediate levels, which were abolished in the L81A/N85A double mutant (Fig. 3c). These binding properties correlated perfectly with their capabilities to activate NF-κB in TLR5^+^ cells in a dose-dependent manner (Fig. 3d) using rFliC as control (Supplementary Fig. 12). Together, these results suggest that CagL mimics the TLR5 activation motif of flagellins. This assumption is further supported by competitive inhibition of rFliC to CagL-TLR5 binding, showing that CagL and FliC compete for the same binding site in TLR5 (Supplementary Fig. 13). Interestingly, TLR5 activation by *Salmonella* FliC involves besides D1 another domain, called D0 (ref. ^12^), however, such a domain is absent in CagL. Thus, CagL represents a unique TLR5-activator protein.

### TLR5-dependent responses in *H.pylori*-infected humans and mice

**Fig. 4:**
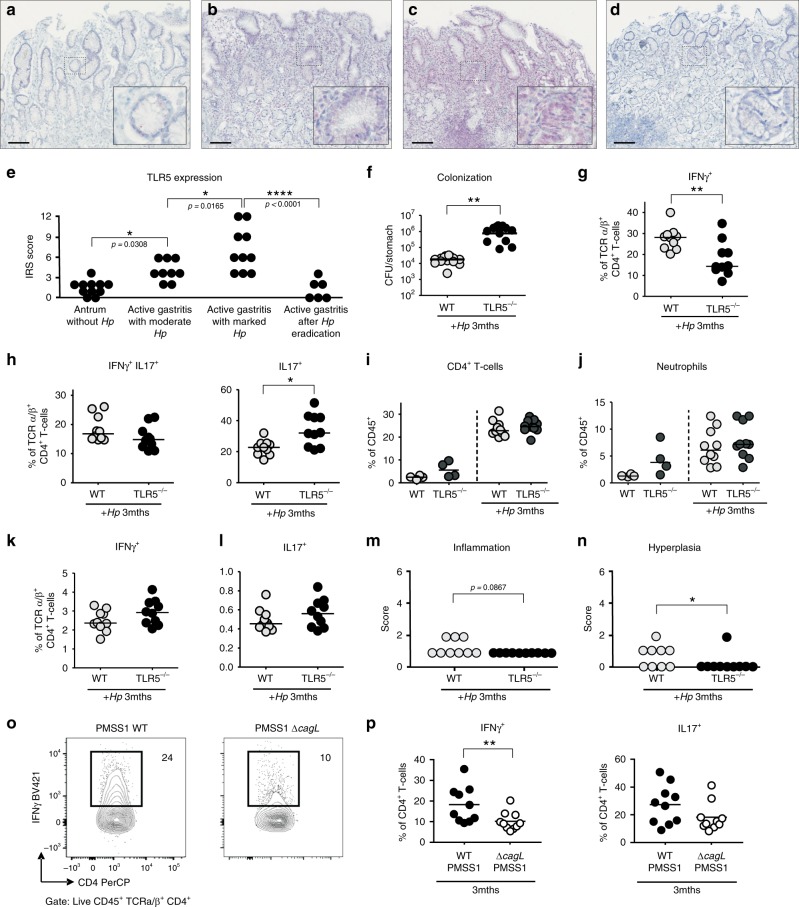
TLR5 responses in the gastric mucosa of *H. pylori-*infected patients and mice. **a**–**d** Immunohistochemistry of TLR5 expression in gastric biopsies of patients. Scale bar: 200 µm. Non-infected antrum mucosa (**a**) exhibits neither pathological changes nor TLR5-positive cells, whereas antrum mucosa (**b**) with moderately chronic, slightly active gastritis and moderate *H. pylori* colonization shows cytoplasmic and nuclear TLR5 staining of the surface epithelium and cytosolic staining of plasma cells. **c** Antrum mucosa with chronic moderately active gastritis in the presence of marked *H. pylori* colonization with a basal lymphoid follicle and slight intestinal metaplasia with slight atrophy of glands shows strong cytoplasmic and nuclear TLR5 staining of the epithelium and cytosolic staining of plasma cells. **d** Antrum mucosa of a gastritis patient who underwent treatment for bacterial eradication. TLR5-expression was reduced to non-infected control levels. **e** ImmunoReactive Score (IRS) quantification of TLR5 staining in patients. *p ≤ 0.05; ****p < 0.0001 (one-way ANOVA). **f**–**n** *tlr5*^−/−^ and wild-type (WT) mice were infected with *H. pylori* for three months (*+Hp* 3mths). **f** Bacterial colonization as determined by plating and colony counting. **g** frequencies of IFN-γ^+^, **h** IL-17^+^ and of cells expressing both cytokines, among gastric lamina propria CD4^+^ TCRα/β^+^ T cells, as determined by flow cytometry. **i**, **j** Frequencies of lamina propria CD4^+^ T cells and Ly6G^+^ neutrophils among all CD45^+^ leukocytes. **k**, **l** Frequencies of IFN- γ ^+^ and IL-17^+^ cells among all MLN CD4^+^ TCR α/β^+^ T cells. **m**, **n** Gastric inflammation and hyperplasia, respectively, as scored on a scale of 0–6 based on the updated Sydney classification on two Giemsa-stained sections per mouse. **o**, **p** WT mice were infected with *H. pylori* PMSS1 WT or its CagL-deficient mutant for three months. Frequencies of IFN-γ^+^ and IL-17^+^ cells, among gastric lamina propria CD4^+^ TCRα/β^+^ T cells, as determined by flow cytometry. The nonparametric Mann–Whitney *U*-test was used for all statistical comparisons (**f**–**p**); *p < 0.05; **p < 0.01; ***p < 0.001. Source data are provided as a Source Data file.

We next examined immunohistochemically if the above findings are of in vivo relevance in patients. Remarkably, while antrum biopsies from healthy non-infected individuals showed no TLR5 staining (Fig. 4a), TLR5 signals appeared upon infection and gradually increased with the grade of *H. pylori* colonization and severity of inflammation in the gastric mucosa as classified by the updated Sydney System^20^ (Fig. 4b, c). TLR5^+^ signals were mainly detected in epithelial cells, plasma cells and granulocytes within the lamina propria, while goblet cells were not stained (Supplementary Fig. 14). Conversely, treatment of *H. pylori* with antibiotics downregulated TLR5 expression in the gastric mucosa to levels detected in uninfected individuals, as assessed at 6–8 weeks after successful eradication (Fig. 4d). Immunoreactive scoring^21^ confirmed that TLR5 is significantly induced by infection and coincides with inflammation in patients (Fig. 4e). We next sought to examine the functional contribution of TLR5 to *H. pylori*-specific immunity in mice. To this end, we infected BL6 mice proficient or deficient for *tlr5* with strain PMSS1 for three months and analyzed bacterial colonization levels, gastric mucosal leukocyte infiltration and pathology, and gastric and lymph node T-cell responses at the study endpoint. *Tlr5*^−/−^ animals exhibited a clear defect in controlling the infection (Fig. 4f), which was associated with strongly reduced Th1 responses in the gastric mucosa (Fig. 4g; Supplementary Fig. 15b). Interestingly, the gastric frequencies of T cells and neutrophils, as well as of Th17 and mixed Th1/Th17 T-cell responses were normal or enhanced in these animals (Fig. 4h-j; Supplementary Fig. 15a, b), as were Th1 and Th17 frequencies in the mesenteric lymph nodes (Fig. 4k, l; Supplementary Fig. 15b). The overall inflammation and gastric pathology, as scored by the updated Sydney system^20^, was similar and slightly reduced, respectively, in *tlr5*^−/−^ relative to WT animals (Fig. 4m, n). No differences in T-cell or neutrophil frequencies (Fig. 4i, j; Supplementary Figs. 15a, 16a, b), or in gastric histopathology (data not shown) were observed in naive *tlr5*^−/−^ relative to WT animals. Interestingly, chronic infection of WT mice with a PMSS1Δ*cagL* mutant phenocopied the effects of TLR5 deficiency with respect to gastric Th1 responses (Fig. 4o, p, Supplementary Fig. 15b). The colonization of this mutant was slightly, but not significantly, reduced (Supplementary Fig. 16c). The combined results suggest that the detection of CagL-positive *H. pylori* by TLR5 is an important element in the generation or maintenance of a local adaptive immune response to this infection, and loss of TLR5 results in hyper-colonization. TLR5 deficiency thus phenocopies the effects of genetic inactivation of the entire T4SS.

### CagL acts as TLR5 agonist for cytokine responses

Finally, we aimed to investigate the role of TLR5 signaling in initiating innate and adaptive immune responses. Gene expression profiling of TLR5^+^ and parental cells exposed to *H. pylori* or not was conducted by microarrays and revealed the significant up-regulation of tumor necrosis factor (TNF) and NF-κB-dependent chemokine responses in TLR5^+^ compared to the parental cells (Supplementary Figs. 17 and 18; Supplementary Tables 1 and 2). In particular, several chemokines and cytokines (CCL2, CCL20, CXCL1, CXCL10, IL-32, IL-17F and others) were differentially expressed upon infection. Interestingly, CCL20 was reported previously to be specifically induced by *Salmonella* flagellin, but not by LPS or intestinal microbiota, for recruitment of immature dendritic cells, which then triggered innate and adaptive immunity^22,23^. We confirmed by ELISA that CCL20 expression was also stimulated in a TLR5-dependent manner in HEK293 reporter cells and cultured T84 cells expressing endogenous TLR5 by co-incubation with rCagL or rFliC, and infection with WT *H. pylori*, but not Δ*cagL* mutant (Supplementary Fig. 19). In addition, CXCL10 was differentially expressed by infected TLR5^+^ cells in our microarrays.

**Fig. 5:**
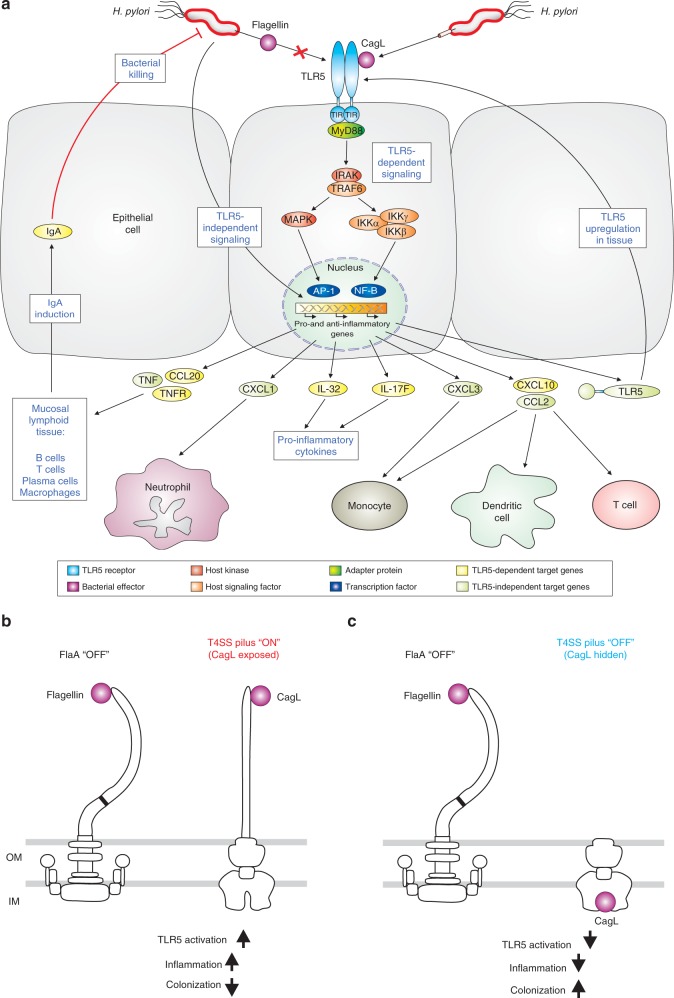
Models for TLR5-mediated and CagL-dependent *H. pylori* pathogenesis. **a** TLR5 is not expressed in healthy gastric tissue. *H. pylori* infection induced the expression of TLR5 in infected gastric patients and TLR5 disappeared after eradication suggesting a tight regulation of this receptor. These data are in agreement with infection studies in WT and TLR5^−/−^ mice, which document that TLR5 controls immune responses and colonization by *H. pylori*. TLR5 recognition of *H. pylori* occurs through the T4SS pilus protein CagL having a D1 mimetic motif, while recognition of flagellin is avoided. In addition, CagL-mediated TLR5 activation up-regulated the expression of several chemokines and cytokines, as well as its receptors. Various immune cell attracting chemokines such as CXCL1, CXCL3, CCL2, CXCL10, CCL20 and cytokines TNF, IL-32 and IL-17F were increased both by TLR5-dependent and -independent signaling pathways as indicated. Moreover, the combined expression of CCL20, TNF and TNF receptor (TNFR) imply the induction of mucosal lymphoid tissue formation through TLR5-dependent signaling. Infected gastric biopsies from patients exhibit an increased expression of TLR5 on plasma cells in the gastric mucosa. This raises the possibility of CagL-TLR5 dependent IgA production in mucosal lymphoid tissues and secretion into the gastric lumen to control the *H. pylori* infection. The various signaling-engaged protein classes are highlighted with different colors as outlined at the bottom. **b** *H. pylori* expresses an unresponsive flagellin (“OFF” status) and instead expresses the T4SS-pilus surface protein CagL, which can trigger TLR5 activation when T4SS pili are expressed (“ON” status). **c** However, T4SS pilus functions can be turned on and off through recombination in T4SS proteins^8^. This shift provides *H. pylori* with the unique ability to avoid permanent TLR5 activation by hiding CagL in the T4SS “OFF” status. We propose that, by engaging TLR5, *H. pylori* may be able to control the host inflammatory response and its own colonization.

Recently, a protective mucosal role of flagellin was reported and that flagellin induced CXCL10, and recombinant CXCL10 protected mice from *Candida albicans* infection^24^. Moreover, CXCL10 expression was also induced by engineered anti-tumor T cells expressing flagellin^25^. Furthermore, CCL2 is induced by *H. pylori* in a TLR5-dependent manner. This is particularly interesting as a functional CCL2/CCR2 axis, driving the recruitment of monocytes and macrophages into the infected mucosa, is required for Th1 immunity to *H. pylori*^26^. The loss of CCR2 phenocopies all aspects (hyper-colonization, reduced Th1 responses but normal Th17 responses) of TLR5 deficiency, indicating that one key consequence of TLR5 signaling in gastric epithelial cells may be the recruitment of these cells and the subsequent initiation of a protective T-cell response (see model in Fig. 5a). We propose that *H. pylori* triggers TLR5 signaling in order to eliminate bacterial competitors in the gastric niche. These findings nicely complement previous work on the role of TLR2 (refs. ^27,28^), TLR9 (ref. ^29^) and TLR10 (refs. ^28,30^) during *H. pylori* infection. The unresponsive flagellin and the shift of recognition by TLR5 to CagL demonstrated here, suggest a remarkable co-evolution of these important factors during the course of *H. pylori* interaction with the host. We propose that this shift endows *H. pylori* with the unique ability to avoid permanent TLR5 activation by exposing or hiding CagL, respectively (Fig. 5b, c), which is in line with earlier observations that pilus function can turn on and turn off through recombination in T4SS protein-coding genes^8^. One consequence of long-term infection with CagL-positive strains, but not CagL-negative strains, is a greatly increased likelihood of serious sequelae, particularly peptic ulcer disease and gastric cancer. According to the background levels produced by *cagL* and other *cag*PAI mutants we also propose that there is at least one more *H. pylori* factor targeting TLR5. Learning how TLR5 signaling by *H. pylori* could be responsible for immune control of the bacteria can teach us as much about human biology as it does about the pathogenesis of these important infections.

## Methods

### *H. pylori* growth, mutagenesis and infection experiments

The *H. pylori* strains employed in this study include the *cag*PAI-positive wild-type (WT) isolates P1, P12, 26695, NCTC11637, G27, N6 and PMSS1 as well as the *cag*PAI-negative WT isolates 1061, Safr7, Ka125 and UH4 (refs. ^11,15–18,22,27,31–33^). Isogenic mutant *H. pylori* were generated by standard gene disruption by insertion procedures using kanamycin or chloramphenicol resistance gene cassettes, respectively^31–34^. Genetic complementation of *cagL* and *flaA* genes was performed in the urease locus on the chromosome using standard procedure^34^. All *H. pylori* strains were grown in thin layers on horse serum agar plates supplemented with vancomycin (10 μg/mL), nystatin (1 μg/mL) and trimethoprim (5 μg/mL) and the mutant strains were selected by adding kanamycin (8 μg/mL) or chloramphenicol (4 μg/mL), respectively. All antibiotics were obtained from Sigma-Aldrich (St. Louis, USA). Incubation of the bacteria was performed at 37 °C for 2 days in an anaerobic jar containing a Campygen gas mix of 5% O_2_, 10% CO_2_ and 85% N_2_ (Oxoid, Wesel, Germany). *H. pylori* grown on agar plates was harvested and resuspended in Phosphate Buffered Saline (PBS, pH 7.4) using a sterile cotton swab (Carl Roth, Karlsruhe, Germany). The bacterial concentration was measured as optical density (OD) at 550 nm using an Eppendorf spectrophotometer. The number of bacteria was also cross-checked with colony-forming units (CFU) grown on horse serum agar plates after serial dilution of the bacterial suspension. The eukaryotic cells grown in medium without antibiotics and antimycotics were infected with *H. pylori* at a multiplicity of infection (MOI) of 5 to 200 for 8 h (Supplementary Fig. 1). Optimal results were obtained using MOI of 25, which were then applied in all subsequent studies. The uninfected mock control cells were incubated with equal amount of PBS.

### Protein expression and purification of recombinant CagL

Recombinant CagL^WT^ and mutants were expressed as C-terminal His-tag fusions in vector pET28a (Novagen, Merck Millipore, Darmstadt, Germany). Site-directed mutagenesis of CagL was done by PCR using pET28a-CagL^WT^ plasmid as DNA template. Amplification was performed in 50 μl reactions containing 2 mM MgCl_2_, 0.2 mM dNTP, 0.2 μM of each primer (Supplementary Table 3) and 0.5 units Phusion High-Fidelity DNA Polymerase (NEB, Ipswich, USA), followed by PCR purification (NucleoSpin Gel and PCR Clean-up, Macherey-Nagel, Düren, Germany), digestion with *Dpn*I (NEB) and ligation using T4 DNA Ligase (NEB). Re-sequencing and Western blotting proved the correct expression of CagL in the resulting plasmids. Purification of the CagL variants was performed by using established methods^11^ including some improvement steps. Briefly, induction of expression was carried out at 16 °C for 20 h. CagL was purified under native conditions by affinity chromatography through HisTrap HP (GE Healthcare, Buckinghamshire, UK), a HiTrap Q HP (GE Healthcare) and then gel filtration through Superdex-75 (GE Healthcare). The apparent size of purified CagL was estimated to be 27 kDa. Purification of CagL was judged to be of >95% homogeneity by SDS-PAGE/Coomassie Blue staining (Supplementary Fig. 11). The folded conformation of the purified CagL proteins was confirmed by circular dichroism using spectropolarimeter. HEK293 parental and TLR5^+^ cells were treated with 5 μg/mL of CagL proteins for 12 h. NF-κB activity was monitored by standard luciferase reporter gene assay.

### Protein expression and purification of rTLR5 from zebrafish

To date, a fusion version of TLR5 is the only available soluble recombinant TLR5 (rTLR5) protein to be an active receptor for flagellin recognition^7^. For rTLR5, the N-terminal fragment of zebrafish TLR5 (amino acid residues 22–390) was fused with the C-terminal fragment of hagfish variable lymphocyte receptor (VLR residues 126–200). In addition, the rTLR5 is appended to the C-terminal thrombin cleavage site, Strep-tag II (WSHPQFEK) and His6 tag to facilitate purification. rTLR5 was expressed and purified by established standard procedure^7^. rTLR5 was secreted in the Hi5 insect cell expression system after infecting with rTLR5 expression baculovirus. rTLR5 was purified using Ni-NTA affinity (Qiagen) and Strep-Tactin affinity chromatography (IBA Lifesciences). The purified rTLR5 was dialyzed against in 20 mM HEPES (pH 7.4), 150 mM NaCl and 1.5 mM β-mercaptoethanol and analyzed by SDS-PAGE.

### CagL peptide SPOT arrays

The CagL peptide arrays were generated by the SPOT-synthesis technique. Briefly, the indicated peptides in Supplementary Fig. 4 were synthesized on an amino-functionalized cellulose membrane using Fmoc/*tert*-butyl chemistry. Each spot consists of 5 nmol peptide. For binding assays, the peptide arrays were blocked overnight at room temperature with 10 mL blocking buffer consisting of 2 × blocking buffer concentrate (Sigma-Aldrich) and 5% (w/v) sucrose in TBS-T (0.02 M sodium phosphate buffer with 0.1 M sodium chloride (pH 7) and 0.05% Tween-20). Five µg/mL recombinant zebrafish TLR protein (His-tagged) in blocking buffer were added to the peptide arrays and incubated for 4 h at room temperature while rotating. Afterwards, the arrays were washed three times with 15 mL of TBS-T buffer and then incubated with 40 μL mouse α-Tetra-His-antibody (Qiagen, cat. no. 34670) in 8 mL blocking buffer resulting in a 1:200 dilution for 2 h at room temperature . Finally, a secondary horseradish peroxidase conjugated α-mouse antibody (Life Technology, Darmstadt, Germany, cat. no. 31446) diluted 1:10,000 in 10 mL blocking buffer was used and antibody detection was performed with the ECL Plus chemiluminescence Western blot kit (GE Healthcare) as described below. The mock control without peptide on the blots revealed no signals, as expected (see Fig. 2b, position C20–25).

### Protein binding and competition assays

The indicated recombinant CagL proteins (1 μg each), FliC protein (1 μg) and FliC peptide as control were immobilized on Dotblots, followed by incubation with 1 μg recombinant TLR5 using the above protocol for peptide SPOT arrays. Site-directed mutagenesis in the D1-motif of CagL was performed^34^ to investigate the importance of single amino acids in TLR5 binding. We also accomplished competition assays by adding growing amounts of rFliC as indicated in the reactions shown in Supplementary Fig. 12, in order to investigate if CagL and FliC compete for the same binding site in TLR5. Bound TLR5 was visualized using α-TLR5 antibodies handled according to our Western blot protocol (see below) and signal intensities were quantified densitometrically.

### Eukaryotic cell culture

The human gastric adenocarcinoma cell line AGS (ATCC CRL-1739) was grown in RPMI-1640 medium, which was supplemented with 10% fetal calf serum (Gibco, Paisley, UK). The human embryonic kidney cells (HEK293 parental, ATCC CRL-1573) and HEK293 cells stably transfected with the pUNOhTLR5 (TLR5^+^) or pUNOhTLR4 (TLR4^+^) constructs (cat. no. 293-htlr5 and no. 293-htlr4a, InvivoGen, San Diego, USA) and T84 cells (ATCC CCL-248) were cultured in Dulbecco’s Modified Eagle Medium (DMEM) containing 4.5 g/L d-glucose, 4 mM l-glutamine, 110 mg/L sodium pyruvate, 10% FBS (Invitrogen, Carlsbad, USA) and were supplemented with 1% antibiotic and antimycotic solution (Sigma-Aldrich) and 10 μg/mL blasticidin (InvivoGen). All cell lines were cultivated at 37 °C in incubators with 5% (v/v) CO_2_ and subcultured in a ratio of 1:3 to 1:5 every 2 to 3 days at a confluence of 70 to 90%. Cells were commonly maintained in 75-cm^2^ tissue culture flasks and seeded into 6- or 12-well plates (Greiner-Bio-One, Germany) before infection.

### Whole genome microarray hybridization

RNA was isolated using the RNeasy Mini Kit (Qiagen) according to the manufacturer’s protocol. The quality of RNAs for microarray analysis was approved by the Agilent 2100 Bioanalyzer platform (Agilent Technologies, Santa Clara, USA). The best quality RNAs were used for further steps. One hundred ng of each total RNA sample was used for the linear T7-based amplification and Cy3-labeled cRNA were prepared using the Agilent Low Input Quick Amp Labeling Kit. Yields of cRNA and the dye-incorporation rate were measured with the ND-1000 Spectrophotometer (NanoDrop Technologies, Wilmington, USA). The hybridization of Cy3-labeled cRNA was performed using the Agilent 60-mer oligo microarray processing protocol using the Agilent Gene Expression Hybridization Kit. Briefly, 0.6 μg Cy3-labeled fragmented cRNA in hybridization buffer was hybridized overnight (17 h, 65 °C) to Agilent Whole Human Genome Oligo Microarrays 8x60K V2 using Agilent’s recommended hybridization chamber and oven. Subsequently, the microarrays were washed once with the Agilent Gene Expression Wash Buffer-1 for 1 min at 25 °C followed by a second wash with preheated Agilent Gene Expression Wash Buffer-2 (37 °C) for 1 min Fluorescence signals of the hybridized Agilent Microarrays were detected using Agilent’s Microarray Scanner System. The Agilent Feature Extraction Software (FES) was used to read out and process the microarray image files. The above procedures were performed at the microarray service centre of Miltenyi Biotec (Bergisch Gladbach, Germany).

### Microarray data analysis

Three replicates were analyzed for each of the sample groups. After background correction of intensity data for all individual microarrays, quantile normalization was conducted between arrays. The normalized intensities were log2-transformed and served as basis for further analysis. One-way ANOVA (*p* ≤ 0.01) was applied among the samples and the differential expression analysis between samples was done using two class unpaired Significance Analysis of Microarrays (SAM) method^35^ with TIGR MeV software version 4.9.0, adjusting delta parameter to ensure the lowest median number of false significant genes (FDR = 0). The data were evaluated using Tukey’s post-hoc test (ANOVA) and Benjamini & Hochberg method (FDR). The derived significant differential gene expression data were used for plotting hierarchical clustering by the method of Euclidean distance, complete linkage. The microarray dataset is available at GEO database through accession number GSE123623.

### Functional classification of differentially expressed genes

DAVID (Database for Annotation, Visualization and Integrated Discovery) v6.8 tool was used to identify the differential expressed genes related to biological pathway or process by selecting Genetic Ontology term GOTERM_BP_FAT and KEGG pathway for functional clustering. The significant highly enriched (Highest stringency, EASE Score >2.0, *p* < 0.001) clusters among genes differentially expressed in *H.* pylori-infected TLR5^+^ (2/31 clusters) and parental cells (4/11 clusters), respectively, were identified.

### Transfection of HEK293 cells and NF-κB luciferase assay

HEK293 parental, TLR5^+^ and TLR4^+^ cells (2 × 10^5^/mL each) were cultured in 12-well plates and transfected with 2 µg of the NF-κB luciferase construct^15^ for 48 h with TurboFect reagent according to the manufacturer’s instructions (Thermo Fisher Scientific, Waltham, USA). Transfected cells were infected with *H. pylori* for 8 h at MOI = 25 or treated with rFliC (100 ng/mL) and analyzed by luciferase assay. Briefly, cells were harvested by passive lysis in lysis buffer (25 mM HEPES, 2 mM EDTA, 20 mM DTT, 5% Glycerol, 1% Triton X-100, pH 7.8). A portion of the lysate was used to measure relative light units (luciferase activity) using the Luciferase Assay buffer (25 mM HEPES, 15 mM MgSO_4_, 5 mM ATP, 0.5 mM d-Luciferin, pH 7.8) in Orion Microplate Luminometer (Titertek Berthold, Pforzheim, Germany). The protein concentration of each lysate was measured with Bradford protein assay reagent (Bradford, Hercules, USA) and equal amount of lysis buffer was used as blank. The relative light units were normalized with protein concentration and the values were plotted as ratio of test versus control samples.

### Enzyme-linked immunosorbent assay

Human T84 cells were reported previously to express endogenous TLR5 that is responsive to *Salmonella* FliC^22^. Thus, we infected T84 cells with *H. pylori* or treated them with recombinant proteins as described above. After 24 h, supernatants were collected and subjected to cytokine ELISA. CCL20 concentrations in the culture medium were determined using the Quantikine ELISA kit and protocol provided by the manufacturer (R&D Systems, USA). Treatment of the cells with 10 ng/mL recombinant TNF served as control (R&D Systems)^36^.

### Inhibition of TLRs by neutralizing antibodies

To further determine the identity of the TLR5 ligand on the HEK293 cells, the cells were investigated in the presence or absence of the α-hTLR5 neutralizing antibody. HEK293 parental and TLR5^+^ cells (2 × 10^5^ per mL of culture) were incubated with α-human TLR5 (α-hTLR5) IgA, which is a neutralizing monoclonal antibody (mAB) to human TLR5, that blocks flagellin-induced cellular activation (InvivoGen, cat. no. maba2-htlr5). The neutralizing ability of α-hTLR5-IgA antibodies was accessed at various concentrations ranging from 10 ng/mL to 1 μg/mL as described by the manufacturer. Incubation was allowed for 1 h, followed by treatment with rFliC or *H. pylori* infection as described above. Inhibition of TLR4 in expressing HEK293 cells was performed with a neutralizing monoclonal antibody (mAB) to human TLR4 using a similar protocol (InvivoGen, cat. no. mabg-htlr4). As a positive control for TLR4 activation in HEK293 cells, we applied *Escherichia coli* LPS (Sigma-Aldrich) and polymyxin B as inhibitor using standard protocols (InvivoGen).

### FlaA and CagL sequence analysis

All protein sequences were obtained from the NCBI GenBank database. Multiple protein sequence alignments, such as in Fig. 2c and Supplementary Fig. 9, were performed by applying Clustal Omega program (version 1.2.4), that uses seeded guide trees and HMM profile-profile techniques to generate alignments between three or more sequences (https://www.ebi.ac.uk/Tools/msa/clustalo/). Sequences were assembled manually and aligned by Clustal Omega using the default parameters. Accession numbers for the sequences used in Fig. 2c are as follows *Helicobacter pylori* CagL (NP_207335.1), *Vibrio anguillarum* FlaA (CDQ50898.1), *Edwardsiella tarda* FliC (AAN52540.1), *Serratia marcescens* FliC (BAO34403.1), *Salmonella enterica* FliC (ATI85802.1), *Helicobacter pylori* FlaA (AAD07667.1), *Campylobacter jejuni* FlaA (CAL35451.1) and *Bartonella bacilliformis* FlaA (ABM44761.1).

### 3D modeling

The structure of *H. pylori* FlaA^*Hp*^ in complex with zebrafish TLR5 (zTLR5) was modeled using the complex of *Salmonella* flagellin with zebrafish TLR5 (PDB: 3V47as template)^7^. Modeling was performed with Modeller 9.16^37^. Structure analysis and visualization were done using Swiss-Model^38^ and RasMol^39^.

### Histology

Biopsies from patients in four groups (non-infected controls *n* = 11, medium grade infection *n* = 9, high grade infection *n* = 10, *H. pylori* eradicated by conventional triple therapy *n* = 6) were fixed in 4% neutral buffered formalin and paraffinized in an increasing series of alcohol and xylene. After manual orientation of the biopsies to allow perpendicular cuts the paraffin blocks were cut into 4 micron thick slides and stained with hematoxylin and eosin. For detection of *H. pylori* a Warthin-Starry Sliver stain was performed. All analyses with human biopsies were reviewed and approved by the FAU Ethics Commission Office in Erlangen/Germany (license 344_16 BC to S.B.).

### Immunohistochemistry

α-TLR5 antibodies (Thermo Fisher Scientific, cat. no. 36–3900) were used to stain specifically cytoplasmatic epithelial and inflammatory cells. For retrieval of antigens, deparaffinised sections were heated in citrate buffer (pH 6.0) using a microwave for 20 min Also, endogenous peroxidase was blocked by 20 min incubation with 0.3% hydrogen peroxidase in absolute methanol. Sections were rinsed by washing buffer and nonspecific binding was blocked by the use of normal serum (Nichirei, Tokyo, Japan). Overnight incubation at 4 °C was carried out for binding of the α-TLR5 primary antibody. Afterwards, 30 min incubation with biotinylated secondary antibody was performed, followed by substrate binding by using streptavidin–biotin–peroxidase method. Counterstaining with haemalaun was done additionally in all cases. For all stains, renal positive controls were performed, and staining was only accepted if evaluable results of controls showed the expected results. If not, staining was repeated until internal controls showed appropriate results. Scoring of positivity was performed blinded by two pathologists according to the ImmunoReactive Score (IRS) system^21^. The percentage of positive cells was divided into five grades: 0–4 (0%, <10%, 10–50%, 51–80%, >80%) multiplied with the grade of intensity (graded from 0 to 3) of the immunohistochemical reaction. Pictures in Fig. 4a–d are shown at 40× magnification.

### *H. pylori* infection of mice

C57BL/6 WT and *tlr5*^−/−^ mice (backcrossed for >10 generations to C57BL/6 background by the vendor) were obtained from the Jackson Laboratory (Bar Harbor, USA). Animals were bred and maintained under specific pathogen-free conditions in accredited animal facilities at the University of Zürich. All animal experimentation was reviewed and approved by the Zürich Cantonal Veterinary Office (licence ZH24/2013 to A.M.). Mice were infected orally on two consecutive days with 10^8^ CFU of *H. pylori* PMSS1 at 6 weeks of age and analyzed at 3 months p.i. Uninfected mice served as controls. Infected WT and *tlr5*^−/−^ females were co-housed to minimize possible confounding effects of putative differences in the microbiota of these animals; unfortunately, this was not possible with males. Data in Fig. 4f are pooled from three independent studies, and data in Fig. 4g–j are pooled from 2 of these 3 studies. Data in Fig. 4k, l is from one large independently conducted study. Separate analysis of the co-housed females only (of which 9 were wild type and 5 were *tlr5*^−/−^) confirmed all trends observed with the entire (mixed) cohort, but some comparisons (histology, Th1 infiltration) did not reach statistical significance due to smaller sample sizes. The difference in colonization levels of co-housed WT and *tlr5*^−/−^ females was statistically significant. The *H. pylori* strain used in this study, PMSS1, is a clinical isolate of a patient with duodenal ulcer and the parental strain of the mouse-derivative Sydney strain 1 (SS1)^22^. *H. pylori* was grown on horse blood agar plates and in liquid culture in an anaerobic jar under microaerophilic conditions as described above^27^. Cultures were routinely assessed by phase contrast microscopy for contamination, morphology and motility. For gastric histopathological analysis of mouse tissue, stomach sections were fixed in 10% neutral-buffered formalin prior to paraffin embedding and Giemsa staining. Two longitudinal sections per mouse spanning the length of the stomach from the forestomach/corpus junction to the antrum/duodenum junction were scored with regard to four histopathological parameters (chronic inflammation, gastric atrophy, intestinal metaplasia, mucus pit cell/epithelial hyperplasia), based on the features described in the updated Sydney classification^20^. Specifically, the definition of scores was as follows for chronic inflammation: 0, none; 1, some infiltrates; 2, mild (few aggregates in submucosa and mucosa); 3, moderate (several aggregates in submucosa and mucosa); 4, marked (many big aggregates in submucosa and mucosa); 5, nearly the entire mucosa contains a dense infiltrate; 6, entire mucosa contains a dense infiltrate and hyperplasia: 0, none; 1, single glands (next to infiltrate); 2, one focal area /1–4 crypts (mild); 3; 1–3 foci; 4, multiple foci; 5, > 50% of glands affected; 6, only few small non-hyperplasic areas. Statistical analysis on all mouse work was performed with Prism 6.0 (GraphPad Software). The nonparametric Mann–Whitney U-test was used for all statistical comparisons. Differences were considered statistically significant when *p* < *0.05*. *p < 0.05, **p < 0.01 and ***p < 0.001.

### Leukocyte isolation from mice

For lamina propria (LP) leukocyte isolation, gastrointestinal tissues were opened longitudinally, washed and cut into pieces. Pieces were incubated in Hanks' balanced salt solution with 10% FCS and 5 mM EDTA at 37 °C to remove epithelial cells. Tissue was digested at 37 °C for 50 min in a shaking incubator with 15 mM HEPES, 500 U/mL of type IV collagenase (Sigma-Aldrich) and 0.05 mg/mL DNase I in RPMI-1640 medium supplemented with 10% fetal bovine serum and 100 U/mL penicillin/streptomycin. Cells were then layered onto a 40/80% Percoll gradient, centrifuged and surface of the cell layer was gently washed in PBS with 0.5% BSA. MLN cell suspensions were prepared by digesting the tissue with 500 U/mL of type IV collagenase in RPMI-1640 medium for 15 min followed by pushing through a cell strainer using a syringe plunger.

### Flow cytometry

For surface staining, cells were stained in PBS with 0.5% bovine serum albumin with a fixable viability dye and a combination of the following antibodies: anti-mouse CD45 (30-F11), Ly6G (1A8), CD4 (RM4–5) and TCRβ (H57–597) (all from BioLegend, San Diego, USA, Supplementary Table 4). Fc block (anti-CD16/CD32, Affymetrix, Santa Clara, USA) was included to minimize nonspecific antibody binding. For intracellular cytokine staining of T cells, cells were incubated for 3.5 h in complete IMDM medium containing 0.1 μM phorbol 12-myristate 13-acetate and 1 μM ionomycin with 1:1,000 Brefeldin A (eBioscience, Vienna, Austria) and GolgiStop solutions (BD Biosciences, Franklin Lakes, USA) at 37 °C in a humidified incubator with 5% CO_2_. Following surface staining, cells were fixed and permeabilized with the Cytofix/Cytoperm Fixation/Permeabilization Solution Kit (BD Biosciences) according to the manufacturer's instructions. Cells were stained for 50 min with antibodies to IL-17A (TC11–18H10.1) and IFNγ (XMG1.2, Biolegend, Supplementary Table 4). Total leukocyte counts were determined by adding countBright Absolute Counting Beads (Life Technologies) to each sample prior to analysis. Samples were analyzed on a LSRII Fortessa (BD Biosciences) or sorted on a FACSAriaIII (BD Biosciences) to a purity of >95%. Analysis was performed using FlowJo software (Tree Star, Ashland, USA).

### Immunoprecipitation

1 × 10^7^ of HEK293 TLR5^+^ cells (mock control, infected with *H. pylori* or treated with rFliC) were washed with cold PBS and lysed for 30 min at 4 °C in lysis buffer (20 mmol/L Tris pH 7.2, 150 mmol/L NaCl, 5 mmol/L ethylenediaminetetraacetic acid, 1% Triton X-100, 10% glycerol, 1 mmol/L Na_3_VO_4_, COMPLETE) as in the established standard protocol^33^. Lysates were precleared with protein G-Sepharose (GE Healthcare) for 2 h at 4 °C. Polyclonal α-TLR5 (2 μg per sample, Supplementary Table 4) was added to the supernatants and incubated overnight at 4 °C. Immune complexes were precipitated by the addition of protein G-Sepharose for 2 h and washed once with lysis buffer and three times with 0.5× PBS. Afterwards, the samples were subjected to SDS-PAGE and Western blotting as described below.

### SDS-PAGE and immunoblot analysis

Infected and control cells were harvested and mixed with equal amounts of 2× SDS-PAGE buffer and boiled for 5 min Proteins were separated by SDS-PAGE on 6–12% polyacrylamide gels and blotted onto PVDF membranes (Immobilon-P, Merck Millipore, Darmstadt, Germany). Before addition of the antibodies, membranes were blocked in TBS-T (140 mM NaCl, 25 mM Tris-HCl pH 7.4, 0.1% Tween-20) with 3% BSA or 5% skim milk for 1 h at room temperature. Phosphorylated and non-phosphorylated CagA proteins were detected by incubation of the membranes with a mouse monoclonal α-phosphotyrosine antibody PY99 diluted 1:1,000 (Santa Cruz, USA, cat. no. sc-7020) and a rabbit polyclonal α-CagA antibody diluted 1:2,000 (Austral Biologicals, San Ramon, USA, cat. no. HPP-5003–9), respectively. The rabbit polyclonal α-Flagellin antibody was reported previously^40^. Rabbit polyclonal α-CagL, α-CagM, α-CagN, α-Cag3 and human GAPDH antibodies were generated by immunizing rabbits against protein-derived peptides (Supplementary Table 4)^11^. Immunization was carried out in accordance with German Tierschutzgesetz and Tierschutz-Versuchsverordnung as implementation of the EU directive 2010/63/EU. The protocol was registered and approved by Landesamt für Landwirtschaft, Lebensmittelsicherheit und Fischerei Mecklenburg-Vorpommern (LALLF M-V, Rostock, Germany). All antibodies were affinity-purified and prepared according to standard protocols by Biogenes GmbH (Berlin, Germany). Polyclonal antibodies recognizing TLR5 were purchased from Santa Cruz (USA). As secondary antibodies, horseradish peroxidase-conjugated α-mouse or α-rabbit polyvalent goat immunoglobulins, respectively, were used (Thermo Fisher Scientific, Massachusetts, USA). Antibody detection was performed with the ECL Plus chemiluminescence Western Blot system for immunostaining (GE Healthcare). Uncropped and unprocessed western blot scans are supplied in the Source Data file.

### Fluorescence labeling of *H. pylori*

*H. pylori* G27 WT and its isogenic Δ*cagL* mutant were cultivated on horse serum GC agar plates as described above. For labeling, the bacteria were harvested and resuspended in phosphate buffered saline (PBS, pH 7.4). The labeling of the bacteria with Fluorescein isothiocyanate (FITC) (Sigma-Aldrich) was performed as in standard protocol^17^. After the labeling process, the bacteria were stored at −80 °C in BHI medium (Oxoid) with 20% glycerol (Carl Roth, Karlsruhe, Germany) until use.

### Real-time adhesion of *H. pylori* by the LigandTracer Green System

The LigandTracer Green instrument (Ridgeview Instruments AB, Uppsala/Sweden) was used to quantify the interaction of *H. pylori* to HEK293 TLR5^+^ and parental cells in real time and under live cell conditions. In order to maintain experimental conditions at 37 °C and 5% CO_2_, the LigandTracer was placed in a Hera Cell 150i CO_2_ incubator (Thermo Fisher). Twenty-four hours before the experiments, 2 x 10^6^ of freshly split HEK293 cells were seeded in a Poly-l-lysine (Sigma-Aldrich) coated Nunclone Surface petridishes (Nunc A/S, Roskilde, Denmark) according to the manufacture’s protocol provided by Ridgeview. The confluence of each cell monolayer was about 90% as confirmed by light microscopy. These plates contain a cell-free area, which was used as the background reference. Before the measurement, the growth media was discarded and the cells were washed three times with fresh DMEM and 10% FCS. Three mL of the same media were added and the plate was placed into the LigandTracer Green apparatus. For the experiment, the “general assay” template was used as provided by the LigandTracer Control software. After a 30 min background measurement, 100 μL of FITC-labeled *H. pylori* G27 WT or G27Δ*cagL* of OD_600_ = 1.0 were added to the cells. The fluorescence intensity of bacterial cells binding to the HEK293 cells was measured in the rotating dish over a time course of 90 min, registered once every 60 s by the fluorescence detector and compared to the reference signal of the opposite cell-free part of the dish.

### Cell binding assay

Infection of parental and TLR5^+^ cells at about 80% confluency was performed at a density of about 3.5 × 10^5^ cells in six‐well plates as reported earlier^18^. Following infection with *H. pylori* in a time course of 2, 4 and 8 h, the cells were washed three times with 2 mL of pre-warmed culture medium without antibiotics to eliminate non-attached *H. pylori*. For counting of cell‐bound bacteria as total colony-forming units (CFU), the cells were incubated for 15 min at 37 °C with 1 mL of PBS buffer and 0.1% saponin. Afterwards, the cell suspensions were collected and serial dilutions incubated on GC agar plates. The number of CFUs was determined by growing the bacteria for 4 days and colony quantification.

### Statistical analysis

All experiments were done at least three times with similar results. All in vitro and human data were evaluated via one-way ANOVA followed by Tukey’s test or via two-way ANOVA and Bonferroni’s multiple comparison test with GraphPad Prism statistical software (version 8.0). Significant difference was defined by *p ≤ 0.05, **p ≤ 0.01, ***p ≤ 0.001 and ****p ≤ 0.0001.

### Reporting summary

Further information on research design is available in the Nature Research Reporting Summary linked to this article.

## Supplementary information

Supplementary Information

Reporting Summary

## Data Availability

The microarray data have been deposited in the Gene Expression Omnibus (GEO) database with accession code GSE123623. Other data generated and analyzed during this study are included in this published article and its Supplementary Information files. The source data underlying Figs. 1, 2a, b, 3b-d, 4e-p and Supplementary Figs. 1–6, 12, 13, 16 and 19 are provided as a Source Data file.

## Source data

Source Data
