# Supplementary material for: T4SS-dependent TLR5 activation by Helicobacter pylori infection
Source: Nat Commun. 2019 Dec 16;10:5717. doi: 10.1038/s41467-019-13506-6 (PMC6915727; doi:10.1038/s41467-019-13506-6)
Supplement: Supplementary file 1 — Supplementary Information [file 41467_2019_13506_MOESM1_ESM.pdf]

Supplementary Information

T4SS-dependent TLR5 activation by *Helicobacter pylori* infection

Pachathundikandi et al.

Correspondence to: Steffen.Backert@fau.de

This PDF file includes:

Supplementary Figs. 1 to 19

Supplementary Tables 1 to 4

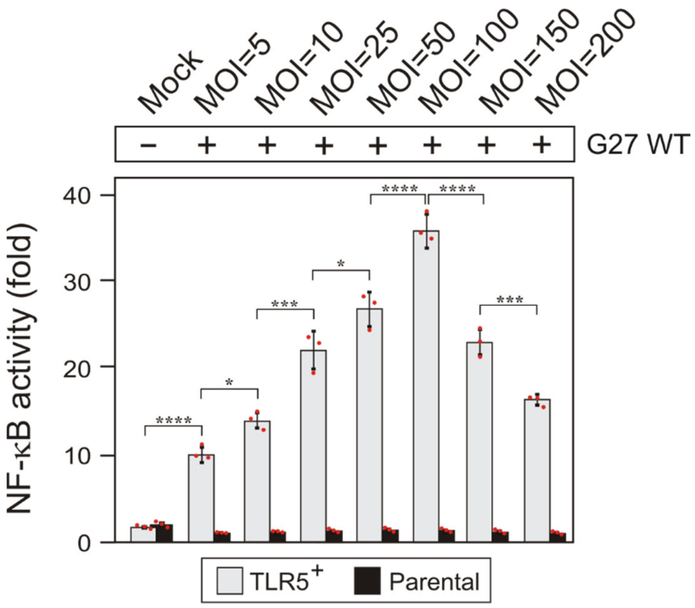

Supplementary Figure 1. Multiplicity of infection (MOI) monitoring of HEK293 control cells (Parental) and HEK293 stably expressing TLR5 (TLR5^{+}). Parental and TLR5^{+} were infected with *H. pylori* strain G27 WT using increasing MOIs of 5, 10, 25, 50, 100, 150 and 200, respectively. NF-κB activation was quantified after 8 hours of infection. The results show that NF-κB activation is growing with increasing MOIs until 100. However, at MOIs of 150 and 200 the cells start to detach from the substratum and NF-κB drop down again. Thus, MOI=25 was used in all subsequent studies. All data are representative as means ± SD of three independent experiments; *, $p \leq 0.05$; ***, $p < 0.001$; ****, $p < 0.0001$ (one-way ANOVA). Each red dot represents a single data point. Source data are provided as a Source Data file.

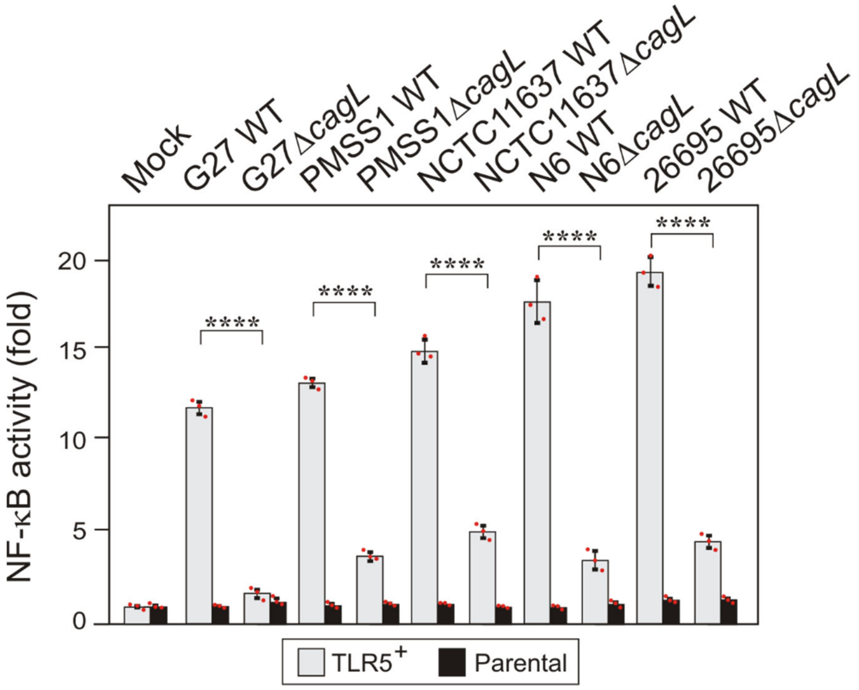

Supplementary Figure 2. CagL-dependent activation of TLR5 on HEK293 reporter cells by multiple *cag*PAI-positive *H. pylori* strains. The indicated clinical type-I isolates exhibit a functional T4SS and the *cagL* gene was inactivated in each strain. TLR5 activation by the WT *H. pylori* strains during infection of parental and $TLR5^+$ cells using NF-κB luciferase reporter gene assays. TLR5 activation was significantly inhibited in the Δ*cagL* mutant infected samples. All results are representative as means ± SD of three independent experiments; ****, $p < 0.0001$ (one-way ANOVA). Each red dot represents a single data point. Source data are provided as a Source Data file.

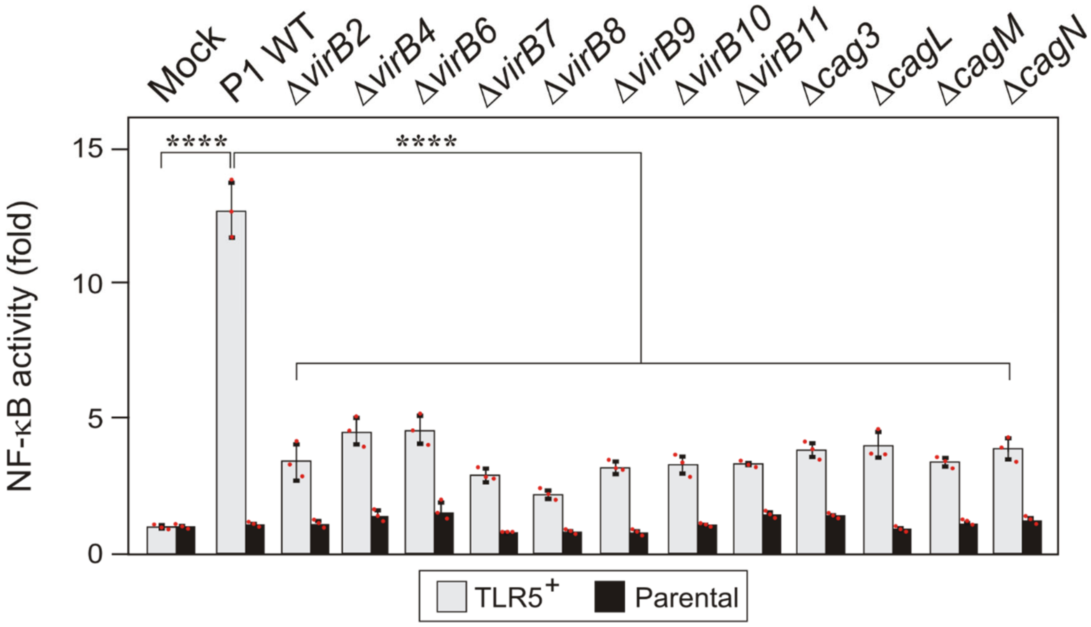

Supplementary Figure 3. Activation of TLR5 by *cag*PAI-positive type-I *H. pylori* requires CagL and other structural T4SS components. The clinical type-I isolate P1 carries a functional T4SS. Besides *cagL*, various other known structural T4SS genes were inactivated by mutagenesis as indicated. TLR5 activation by WT *H. pylori* was significantly downregulated by all shown Δ*virB* and Δ*cag* mutants during infection of parental and $TLR5^+$ reporter cells using NF-κB luciferase reporter gene assays. All data are representative as means ± SD of three independent experiments; ****, $p < 0.0001$ (one-way ANOVA). Each red dot represents a single data point. Source data are provided as a Source Data file.

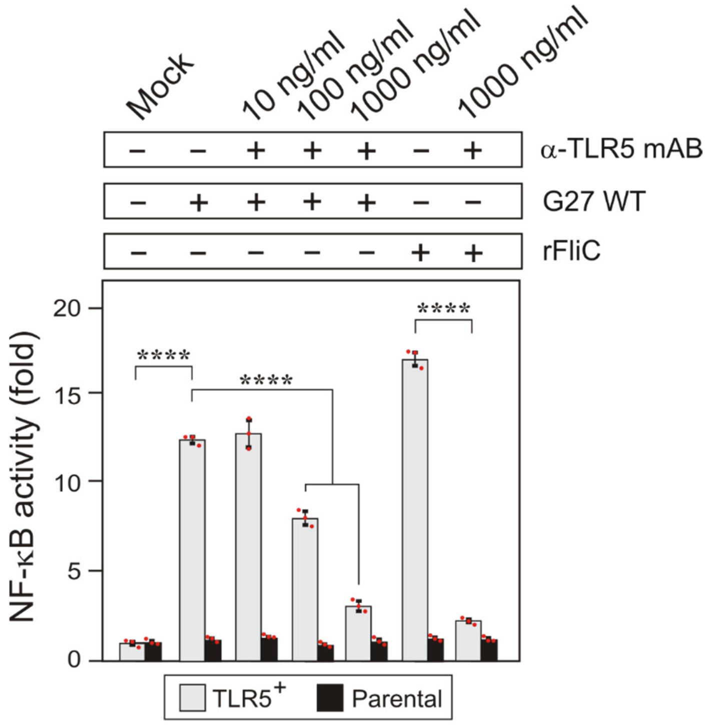

Supplementary Figure 4. Specific blocking of *H. pylori* infection- and *Salmonella* FliC-induced NF-κB stimulation in HEK293 TLR5 reporter cells in the presence of α-hTLR5 neutralizing antibody. HEK293 control and $TLR5^+$ cells were infected with G27 WT *H. pylori* or treated with recombinant *Salmonella* FliC (rFliC) protein as indicated. Growing concentrations of α-hTLR5 neutralizing antibodies were added to the samples in lanes 3-5 and 7, respectively. *H. pylori*- and FliC induced TLR5 activation was measured using NF-κB luciferase reporter gene assays. *H. pylori*- or rFliC-induced NF-κB activation was inhibited by the antibody in a dose-dependent manner. All data are representative as means ± SD of three independent experiments; ****, $p < 0.0001$ (one-way ANOVA). Each red dot represents a single data point. Source data are provided as a Source Data file.

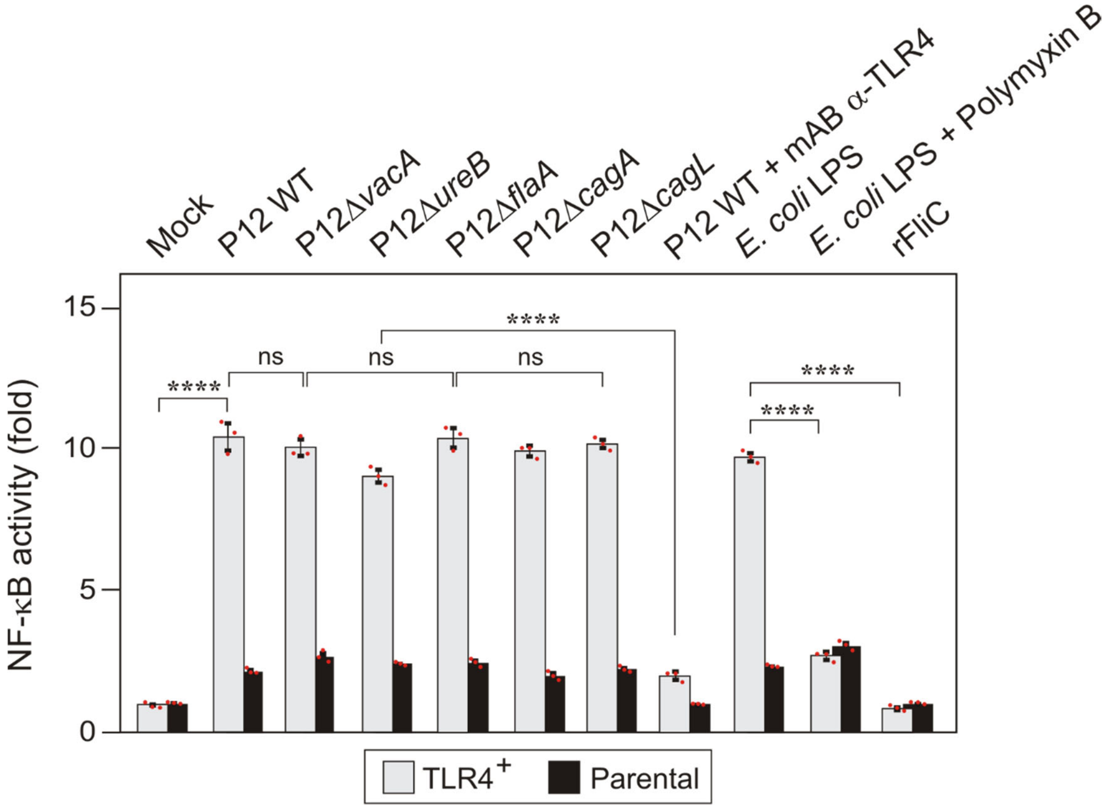

Supplementary Figure 5. Control experiments showing the activation of TLR4 by *H. pylori* WT and isogenic mutant strains in HEK reporter cells. TLR4 activation as quantified by NF-κB luciferase reporter gene assay by all indicated strains in $TLR4^{+}$ cells, but not parental cell controls. TLR4 activation in HEK293 cells does require functional *cagL* or other indicated virulence genes. The α-hTLR4 neutralizing antibodies were added to the WT sample leading to inhibition of NF-κB activation. *E. coli* LPS was added as positive control for TLR4 activation, which was inhibited in the presence of polymyxin B. Recombinant flagellin of *Salmonella* (rFliC) was used as negative control. All data are representative as means ± SD of three independent experiments; ns: not significant; ****, $p < 0.0001$ (one-way ANOVA). Each red dot represents a single data point. Source data are provided as a Source Data file.

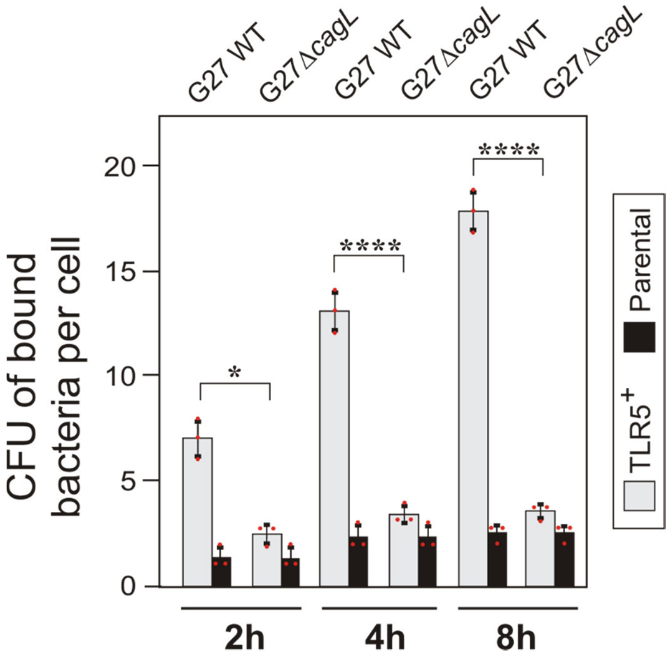

Supplementary Figure 6. Cell binding assay of *H. pylori* infected HEK reporter cells. HEK293 control and TLR5^{+} cells were infected with G27 WT *H. pylori* or isogenic Δ*cagL* mutant. Bacterial cell binding was monitored over 2, 4 and 8 hours by quantifying colony forming units (CFU) per cell as described in Methods.[1] All data are representative as means ± SD of three independent experiments; *, $p \leq 0.05$; ****, $p < 0.0001$ (Student's t-test). Each red dot represents a single data point. Source data are provided as a Source Data file.

a	b	c
1-EDITSGLKQLDSTYQ	1-EDVIFKTYMSSPELL	1-ASLITASQTLVESLK
2-TSGLKQLDSTYQETN	2-IFKTYMSSPELLLTY	2-ITASQTLVESLKNKN
3-LKQLDSTYQETNQQV	3-TYMSSPELLLTYMKI	3-SQTLVESLKNKNFIK
4-LDSTYQETNQQVLKN	4-SSPELLLTYMKINPL	4-LVESLKNKNFIKGIR
5-TYQETNQQVLKNLDE	5-ELLLTYMKINPLDQN	5-SLKNKNFIKGIRKLM
6-ETNQQVLKNLDEIFS	6-LTYMKINPLDQNTAE	6-NKNFIKGIRKLMLAH
7-QQVLKNLDEIFSTTS	7-MKINPLDQNTAEQQC	7-FIKGIRKLMLAHNKV
8-LKNLDEIFSTTSPSA	8-NPLDQNTAEQQCGIS	8-GIRKLMLAHNKVFLN
9-LDEIFSTTSPSANNE	9-DQNTAEQQCGISDKV	9-KLMLAHNKVFLNYLE
10-IFSTTSPSANNEMGE	10-TAEQQCGISDKVLVL	10-LAHNKVFLNYLEELD
11-TTSPSANNEMGEEDA	11-QQCGISDKVLVLYCE	11-NKVFLNYLEELDALE
12-PSANNEMGEEDALNI	12-GISDKVLVLYCEGKL	12-FLNYLEELDALERSL
13-NNEMGEEDALNIKKA	13-DKVLVLYCEGKLKIE	13-YLEELDALERSLEQS
14-MGEEDALNIKKAAIA	14-LVLYCEGKLKIEQEK	24-ELDALERSLEQSKRQ
15-EDALNIKKAAIALRG	15-YCEGKLKIEQEKQNI	15-ALERSLEQSKRQYLQ
16-LNIKKAAIALRGDLA	16-GKLKIEQEKQNIRER	16-RSLEQSKRQYLQERQ
17-KKAAIALRGDLALLK	17-KIEQEKQNIRERLET	17-EQSKRQYLQERQSSK
18-AIALRGDLALLKANF	18-QEKQNIRERLETSLK	18-KRQYLQERQSSKIIV
19-LRGDLALLKANFEAN	19-QNIRERLETSLKAYQ	19-RQYLQERQSSKIIVK
20-DLALLKANFEANELF	20-RERLETSLKAYQSNI	20-Mock control
21-LLKANFEANELFFIS	21-LETSLKAYQSNIGGT	21-Mock control
22-ANFEANELFFISEDV	22-SLKAYQSNIGGTASL	22-Mock control
23-EANELFFISEDVIFK	23-AYQSNIGGTASLITA	23-Mock control
24-ELFFISEDVIFKTYM	24-SNIGGTASLITASQT	24-Mock control
25-FISEDVIFKTYMSSP	25-GGTASLITASQTLVE	25-Mock control

d

Signal peptide | **Mature protein**

MKTLVKNTISSFLLLSVLMA | EDITSGLKQLDSTYQETNQQVLKNLDEIFSTTSPSANNEMGEEDALNIKKAAI
ALRGDLALLKANFEANELFFISEDVIFKTYMSSPELLLTYMKINPLDQNTAEQQCGISDKVLVLYCEGKLKIE
QEKQNIRERLETSLKAYQSNIGGTASLITASQTLVESLKNKNFIKGIRKLMLAHNKVFLNYLEELDALERSLE
QSKRQYLQERQSSKIIVK

Supplementary Figure 7. Generation and design of CagL peptide arrays. Overlapping linear 15-mer peptides derived from the CagL sequence from amino acid position 21-237 (covering the mature CagL protein). Altogether 69 peptides were chemically synthesized on a cellulose membrane by the SPOT method. These peptides were spotted in three lanes on the membrane, corresponding to the sequences shown in panels **a-c**. As indicated here and in Figure 2b, adjacent peptides have an overlap of 12 amino acids and a shift of 3 amino acids from the N- to the C-terminus along the protein sequence. Due to the size of CagL, the positions 20-25 in panel C remained empty and served as the mock control without peptide (white box). **d,** Full-length CagL protein sequence of strain 26695 (NP_207335.1) composed of the cleaved-off signal peptide (position 1-20, light grey box) and mature protein (position 21-237, dark grey box).

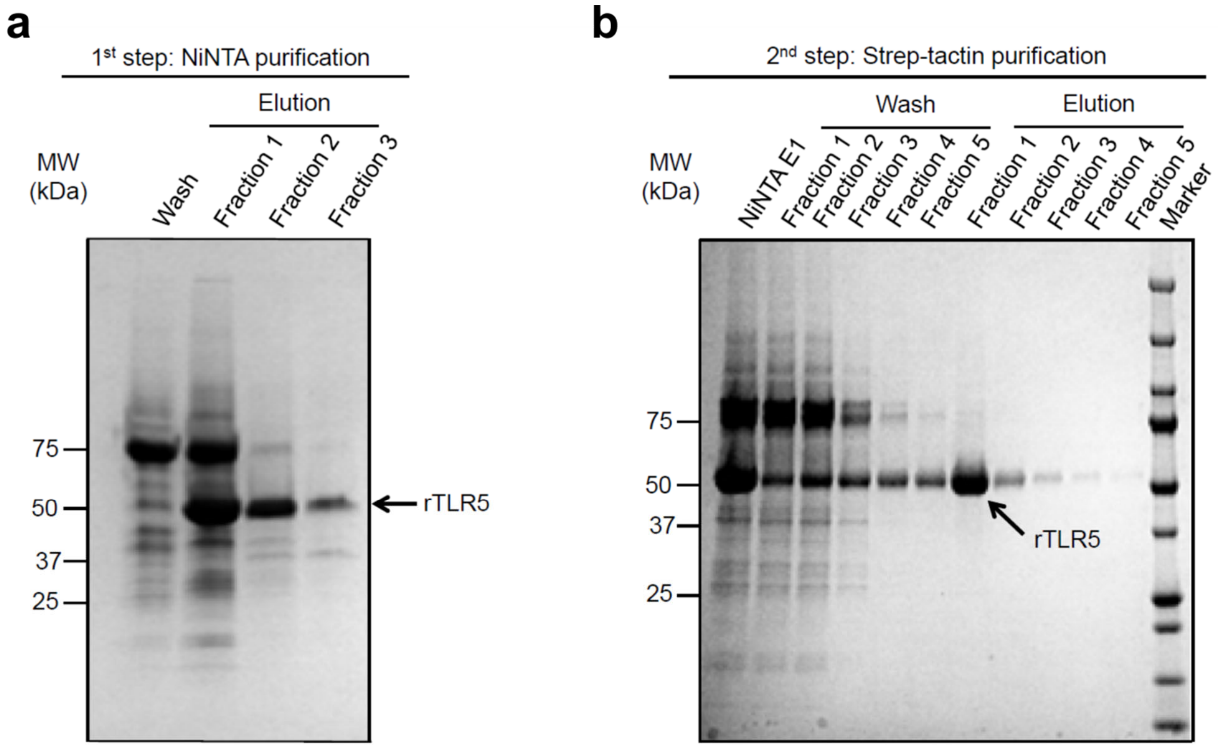

Supplementary Figure 8. Purification and SDS-PAGE analysis of recombinant zebrafish TLR5 (rTLR5). The rTLR5 protein was purified in two steps using NiNTA affinity (**a**) and Strep-tactin affinity (**b**) chromatography. In each step, flow-through fractions of the column were collected and analyzed by SDS-PAGE. The gels were stained with coomassie brilliant blue to visualize protein bands. The arrow indicates the protein band for the rTLR5. **a,** 1st step of purification by Nickel affinity chromatography. rTLR5 was expressed with C-terminal hexa-histidine (His6) and Strep tags and bound to nickel-agarose (Nickel nitrilotriacetic acid, NiNTA) and Strep-tactin affinity columns, respectively. Unbound proteins from the NiNTA column were labeled as “Wash”. The bound protein to the NiNTA was competitively eluted off by addition of imidazole and collected in “Fractions 1-3”. Fraction 1 contained rTLR5 but also other cellular proteins. **b,** 2nd step of Strep-tactin purification. Due to impurities after the 1st step purification, the elution fraction from the NiNTA (NiNTA E1) was applied to Strep-tactin column. Some amount of rTLR5 was lost during washing off the unbound proteins (Wash Fractions 1-5). Elution was done by addition of desthiobiotin and flow-through was collected as Elution Fraction 1-5. Fractions to contain the rTLR5 were pooled.

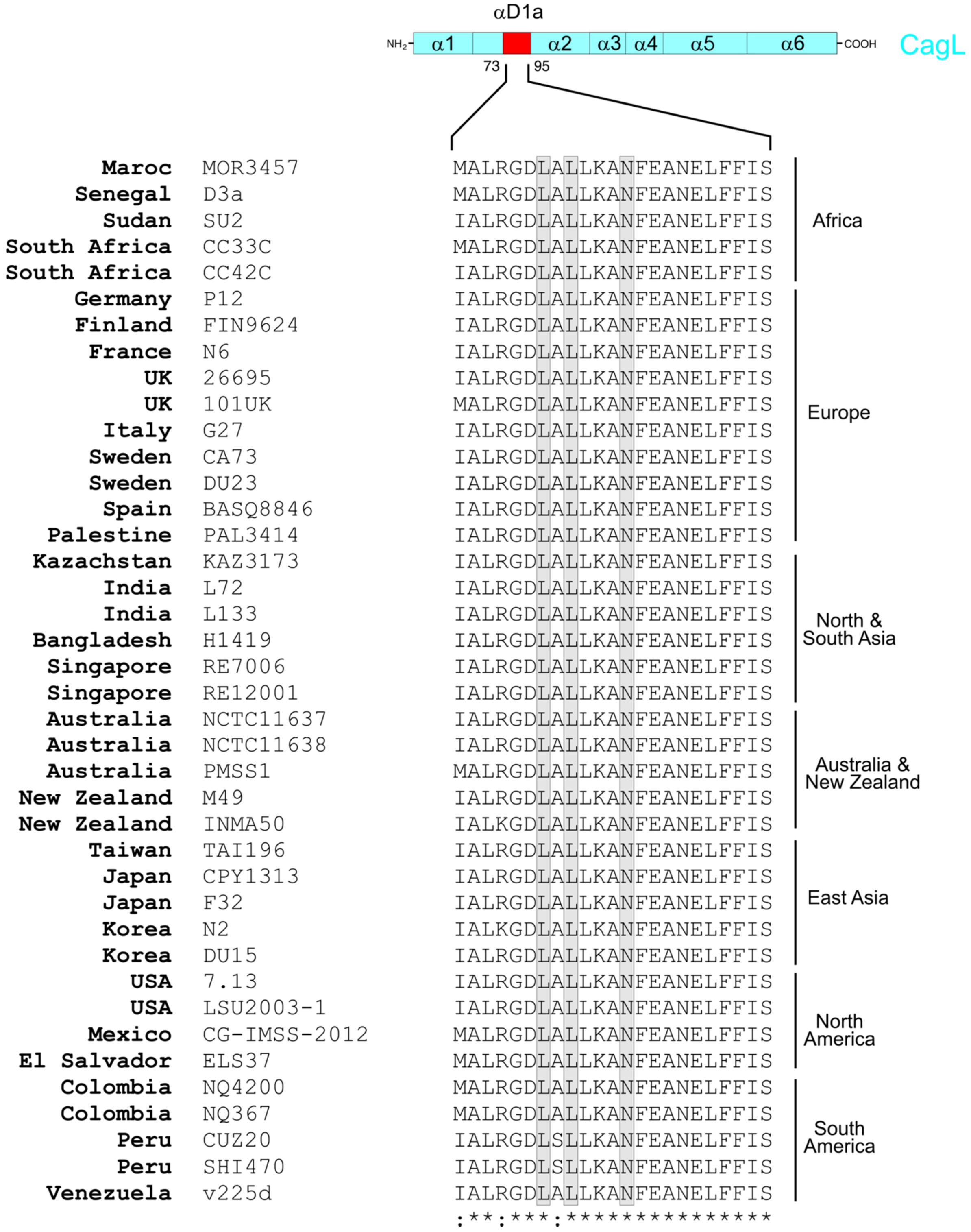

Supplementary Figure 9. The CagL D1 motif mimetic sequence is conserved in worldwide *H. pylori* strains. The CagL protein sequences were obtained from databases and multiple sequence alignment of the D1 motif was performed by using the Clustal Omega program

(https://www.ebi.ac.uk/Tools/msa/clustalo/). The accession numbers for CagL are as follows: MOR3457 (CBV36450.1), D3a (CBV36023.1), SU2 (CBV36656.1), CC33C (CBV35959.1), CC42C (CBV35993.1), P12 (ACJ07700.1), FIN9624 (CBV36082.1), N6 (unpublished), 26695 (NP_207335.1), 101UK (CBV35899.1), G27 (ACI27259.1), CA73 (AAR03901.1), DU23 (AAR03962.1), BASQ8846 (CBV35929.1), PAL3414 (CBV36538.1), KAZ3173 (CBV36246.1), L72 (CBV36334.1), L133 (CBV36363.1), H1419 (CBV36111.1), RE7006 (CBV36597.1), RE12001 (CBV36625.1), NCTC11637 (ADF42575.1), NCTC11638 (AAC44697.1), PMSS1 (OWT35127.1), M49 (CBV36421.1), INMA50 (CBV36189.1), TAI196 (CBV36685.1), CPY1313 (EJB15505.1), F32 (BAJ58097.1), N2 (CBV36479.1), DU15 (CBV36053.1), 7.13 (WP_000855271.1), LSU2003-1 (CBV36392.1), CG-IMSS-2012 (ERM21230.1), ELS37 (AFF20349.1), NQ4200 (EJB29468.1), NQ367 (CBV36508.1), CUZ20 (ADO03756.1), SHI470 (ACD48274.1) and v225d (CBV36716.1). Three amino acid residues (L79, L81, N85) constituting the LxLxxxN motif, which can interact with and activate TLR5, are marked with grey boxes and shown to be conserved in all CagL proteins investigated so far.

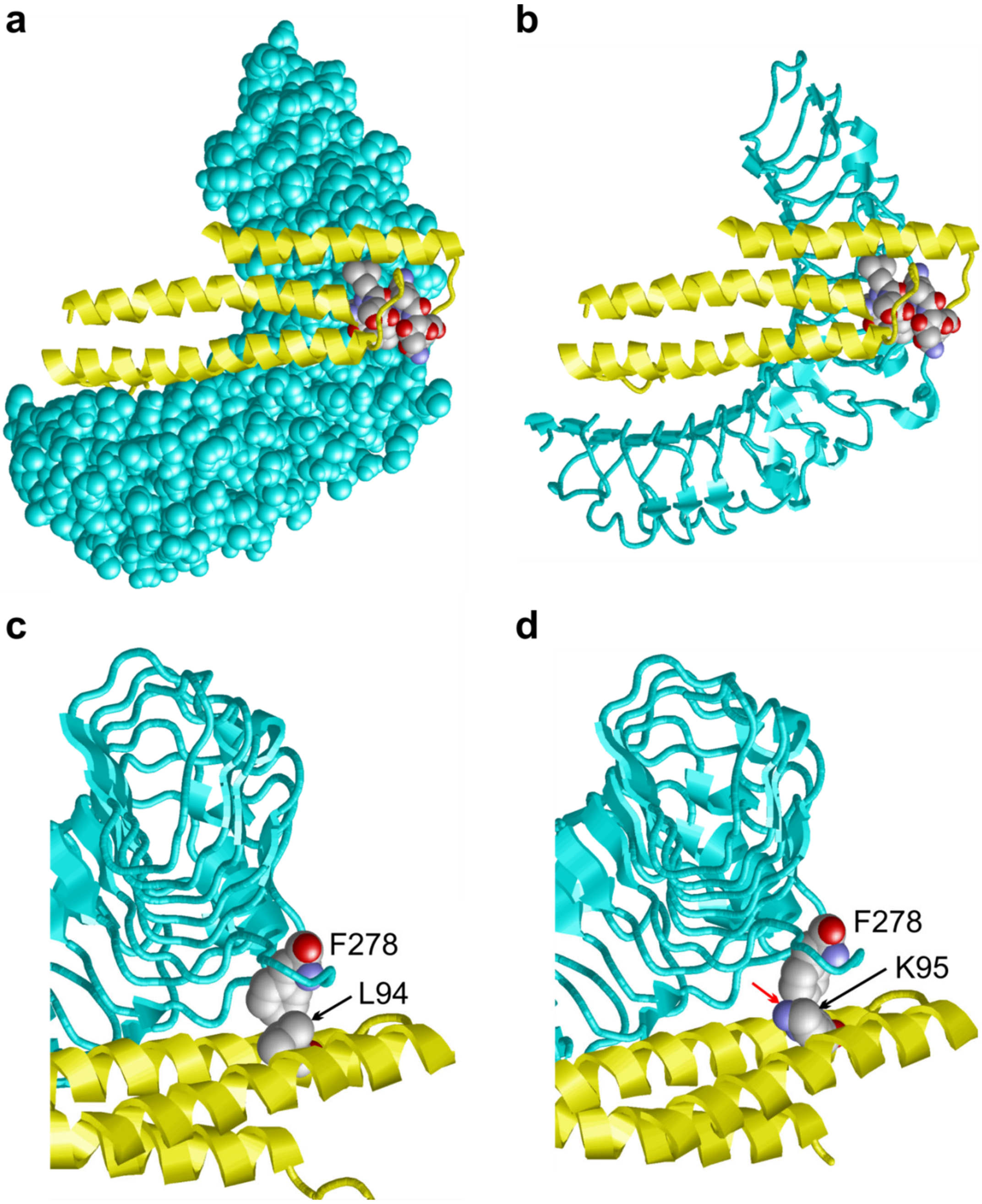

Supplementary Figure 10. Structural properties of the flagellin-TLR5 complex. a, Crystal structure of *Salmonella* flagellin (FliC, yellow, residues 129-409 omitted for clarity) in complex with zebrafish TLR5 (cyan) (PDB:3V47).[2] Residues constituting the conserved binding motif of FliC are represented in space-filled presentation and colored by atom-types. **b,** Same presentation as in (**a**) but with TLR5 in ribbon presentation (cyan). **c,** Close-up view highlighting the hydrophobic interaction between L94 (*Salmonella* FliC) and F278 of TLR5. **d,** Same view as in (**c**) showing the modeled interaction between FlaA^{Hp} and TLR5. In the complex model, a positively charged lysine would be placed in the proximity of the hydrophobic F278 sidechain, thus resulting in an unfavorable interaction (red arrow).

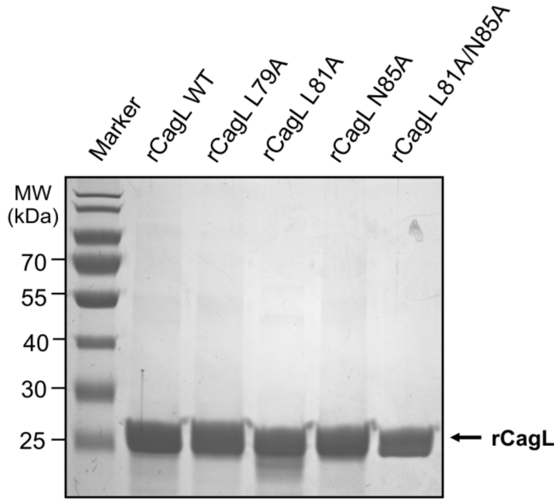

Supplementary Figure 11. Site-directed mutagenesis in the D1-motif of CagL and purification of the recombinant proteins. Coomassie-stained gel showing purified recombinant CagL (rCagL) proteins of 2 µg each of rCagL WT and rCagL with point mutations at L79A, L81A, N85A or L81A/N85A, respectively. The apparent size of purified rCagL variants is about 27 kDa (arrow). The results revealed that rCagL was purified to >95% homogeneity.

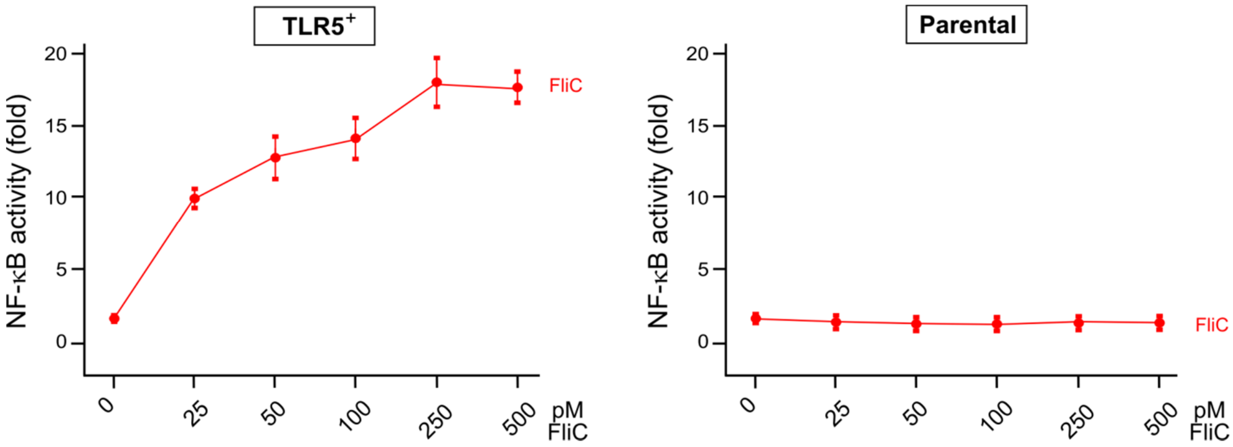

Supplementary Figure 12. Dose-response curves of NF-κB activity in TLR5^{+} and parental HEK293 cells treated with the indicated amounts of *Salmonella* FliC protein. EC_{50} value of FliC was determined to be 27.5 pM by using AAT Bioquest (https://www.aatbio.com). Source data are provided as a Source Data file.

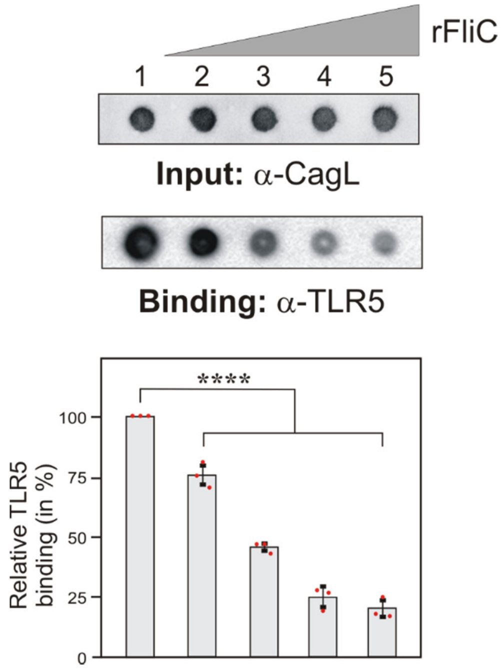

Supplementary Figure 13. Binding of recombinant rTLR5 to CagL can be inhibited by recombinant FliC in a dose-dependent manner. Recombinant CagL (1 µg) was immobilized on Dotblots, followed by incubation with recombinant TLR5 (1 µg) in a standard protein binding assay. rFliC protein was added in increasing amounts of 100 ng (lane 2), 250 ng (lane 3), 500 ng (lane 4) and 1 µg (lane 5). The results revealed a dose-dependent inhibition of TLR5 binding to CagL. All data are representative as means ± SD of three independent experiments; ****, $p < 0.0001$ (one-way ANOVA). Each red dot represents a single data point. Source data are provided as a Source Data file.

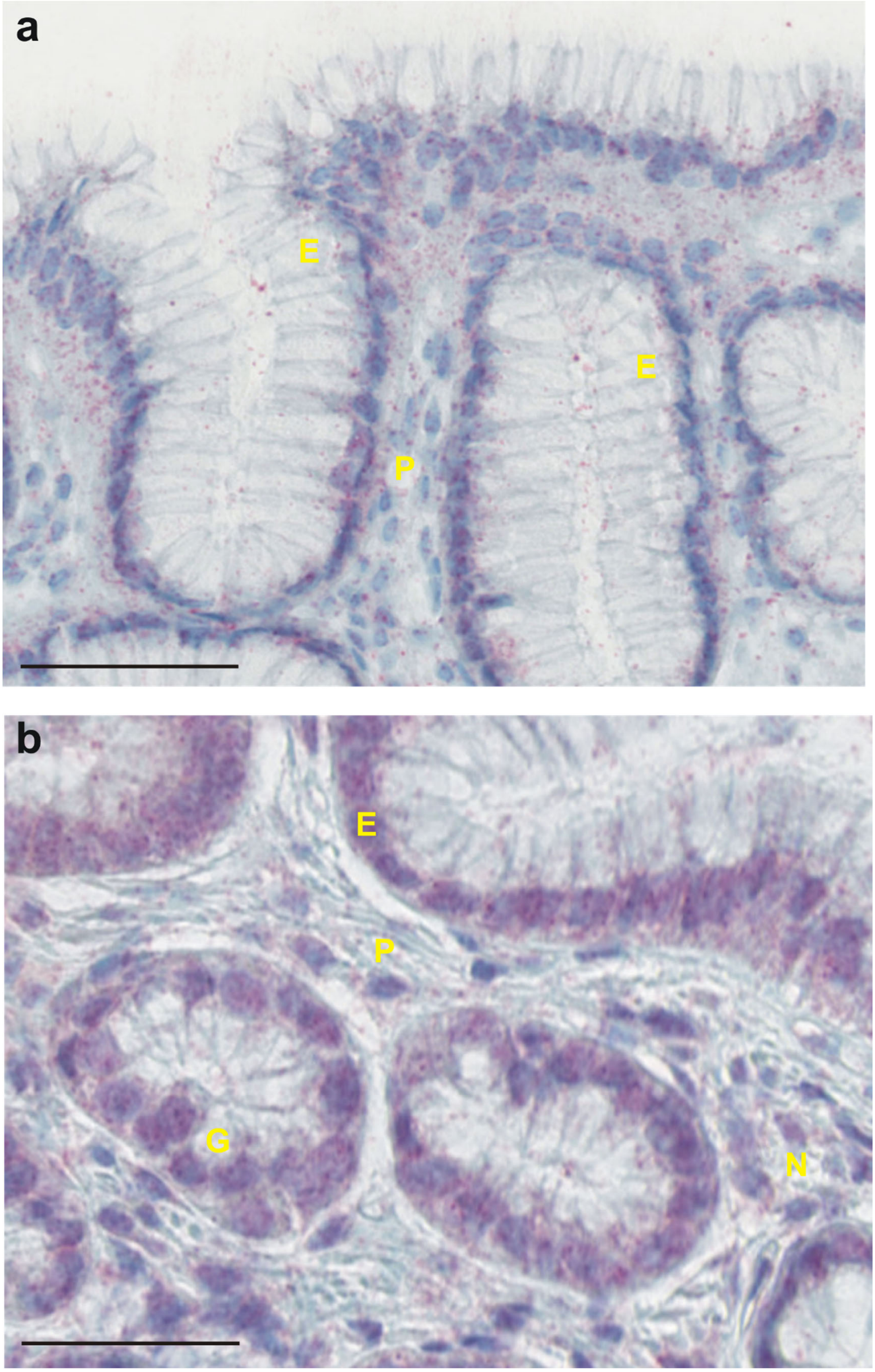
a
E
E
P
b
E
P
G
N

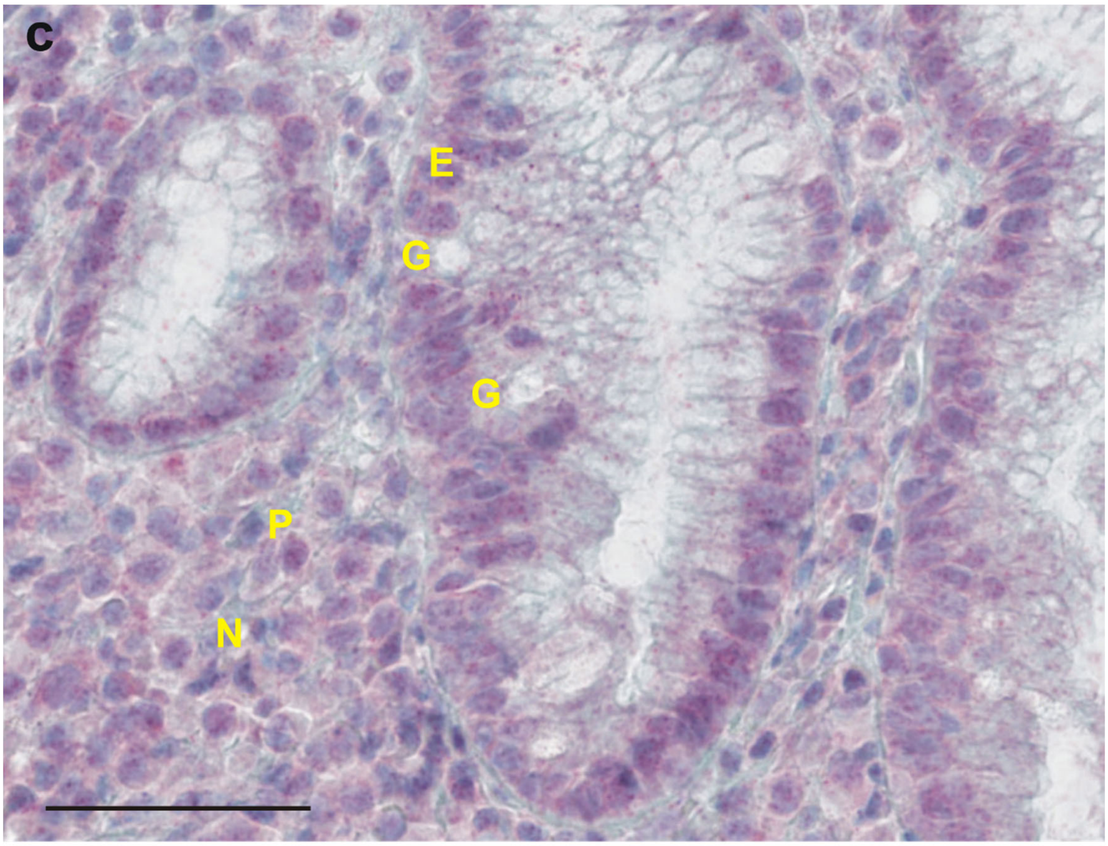
c
E
G
G
P
N

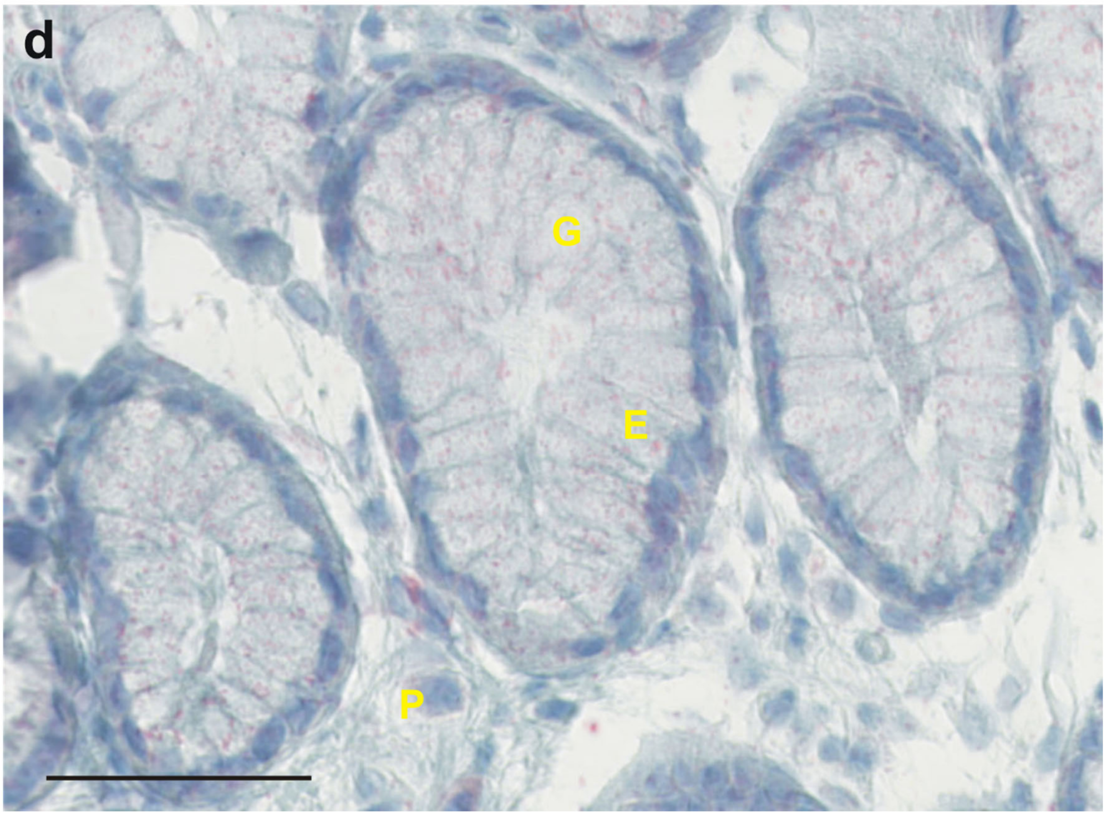
d
G
E
P

Supplementary Figure 14. Enlarged sections of TLR5 immunohistochemistry in *H. pylori* infected patients. Expression of TLR5 in gastric biopsies of human patients: **a,** Non-infected antrum mucosa; **b**, Antrum mucosa with moderately chronic, slightly active gastritis and moderate *H. pylori* colonisation; **c,** Antrum mucosa with chronic moderately active gastritis in the presence of marked *H. pylori* colonisation; **d**, Antrum mucosa of a gastritis patient who underwent treatment for bacterial eradication. Enlarged sections of hemalaun stainings are shown at high magnification (200x). Examples of epithelial cells (E), plasma cells (P), neutrophils (N), goblet cells (G) are marked with yellow letters. Scale bar: 100 µm.

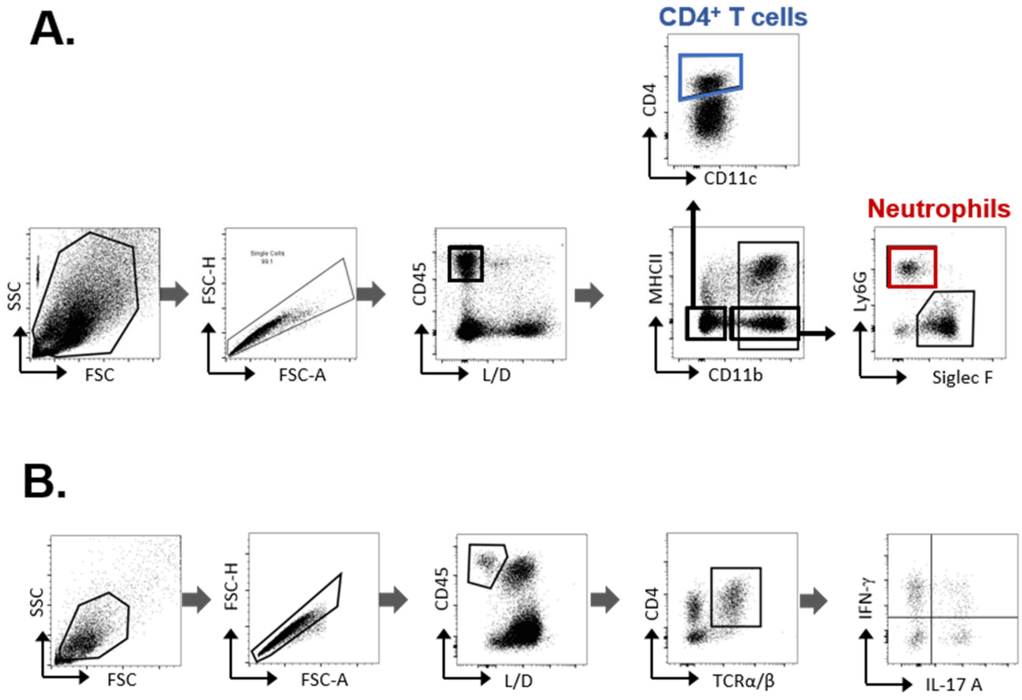

Supplementary Figure 15. Gating strategies used for cell sorting. a, Gating strategy used for the identification of neutrophils (live, single, CD45+ CD11b+ MHCII- Ly6G+) and CD4+ T cells (live, single, CD45+ CD11b- MHCII- CD4+) in the gastric lamina propria (Fig.4 i,j; Supplementary Figure 16a,b). **b,** Gating strategy used for the identification of IFN-γ+ and IL-17+ CD4+ T cells (live, single, CD45+ TCRα/β+ CD4+) and in the gastric lamina propria (Fig.4 g,h,o,p) and MLNs (Fig.4 k,l).

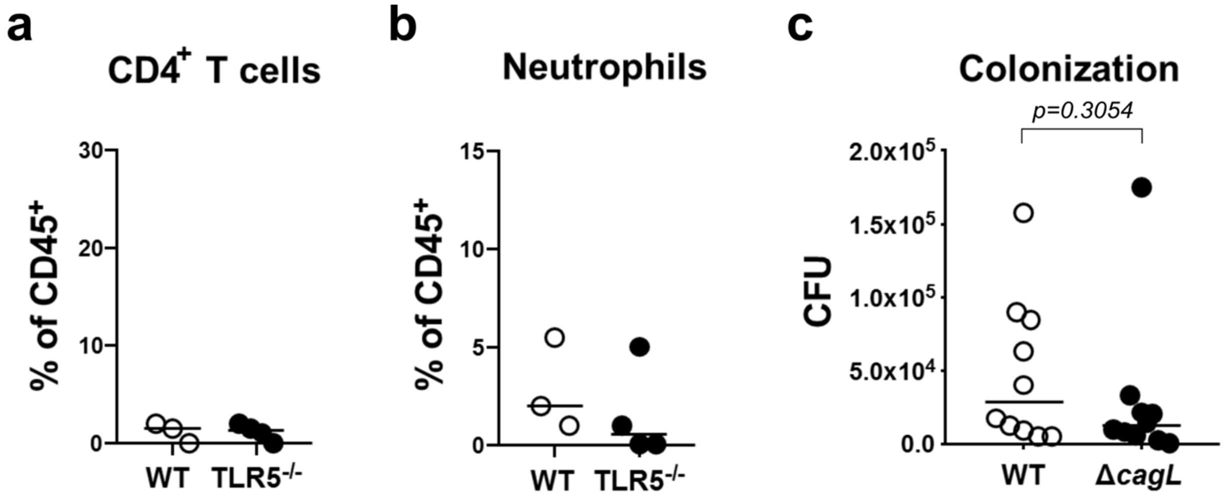

Supplementary Figure 16. Impact of the loss of TLR5 expression on T cells, neutrophils and *H. pylori* colonization in mice. a, b, The loss of TLR5 expression does not change the infiltration of $CD4^+$ T cells and of neutrophils into the gastric mucosa. **c,** The colonization of the PMSS1 *cagL* mutant was slightly, but not significantly reduced. Therefore, the difference in immunological responses exhibited in mutant-infected relative to WT-infected mice likely is due to reduced CagL-driven TLR5 signaling. Source data are provided as a Source Data file.

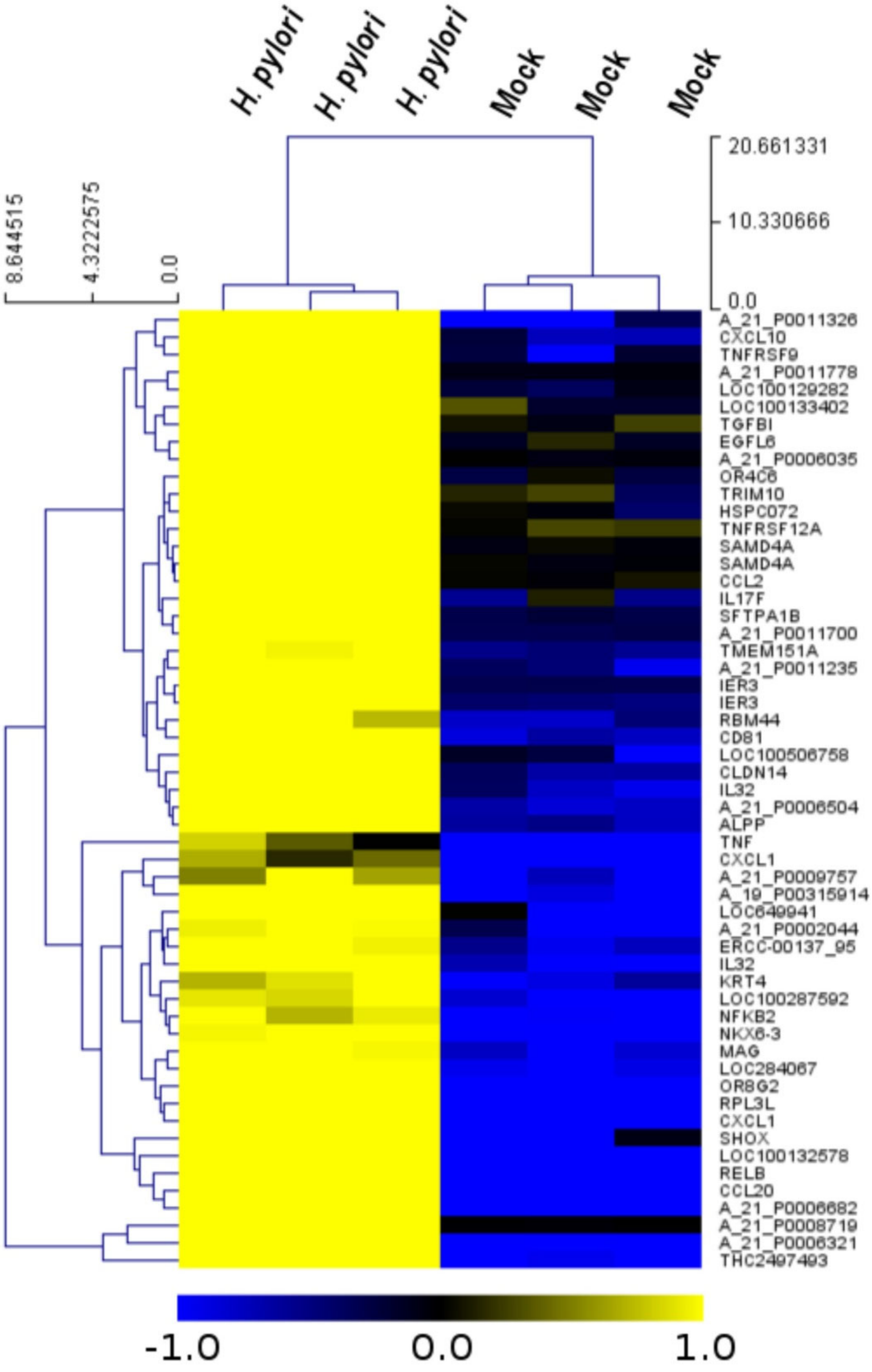

Supplementary Figure 17. Hierarchical cluster map of major differentially expressed genes in TLR5+ cells as analysed by whole genome microarrays. The significant differential gene expression data sets were obtained by SAM analysis in TIGR MeV software. The intensity of the coloured regions was proportional to abundance of transcript expression normalized to the intensity range of -1 to 1. The clustering was performed using Euclidean distance, complete linkage method for the different genes on left and between samples on the top.

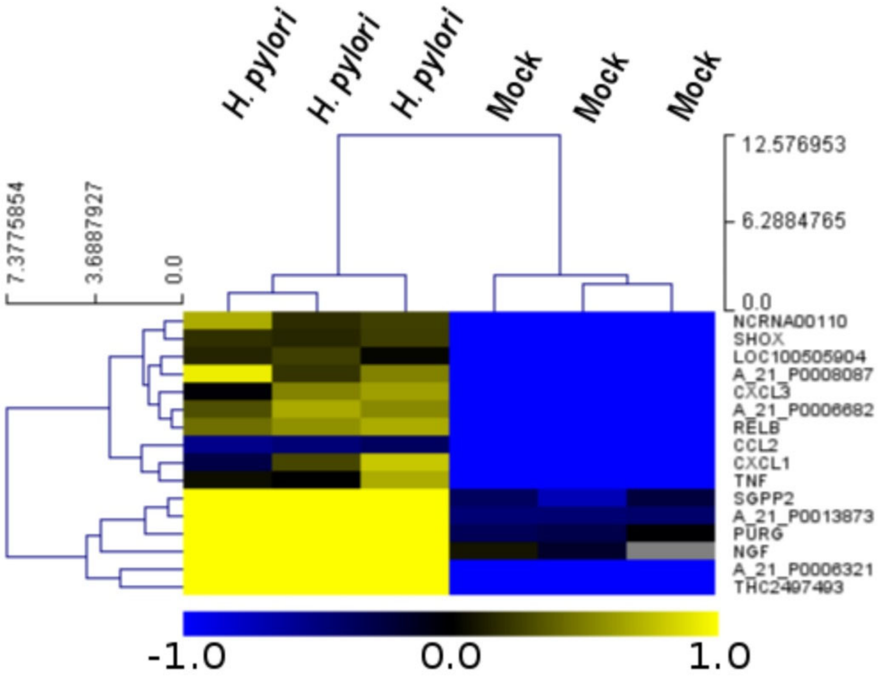

Supplementary Figure 18. Hierarchical cluster map of major differentially expressed genes in parental cells as analysed by whole genome microarrays. The significant differential gene expression data sets were obtained by SAM analysis in TIGR MeV software. The intensity of the coloured regions was proportional to abundance of transcript expression normalized to the intensity range of -1 to 1. The clustering was performed using Euclidean distance, complete linkage method for the different genes on left and between samples on the top.

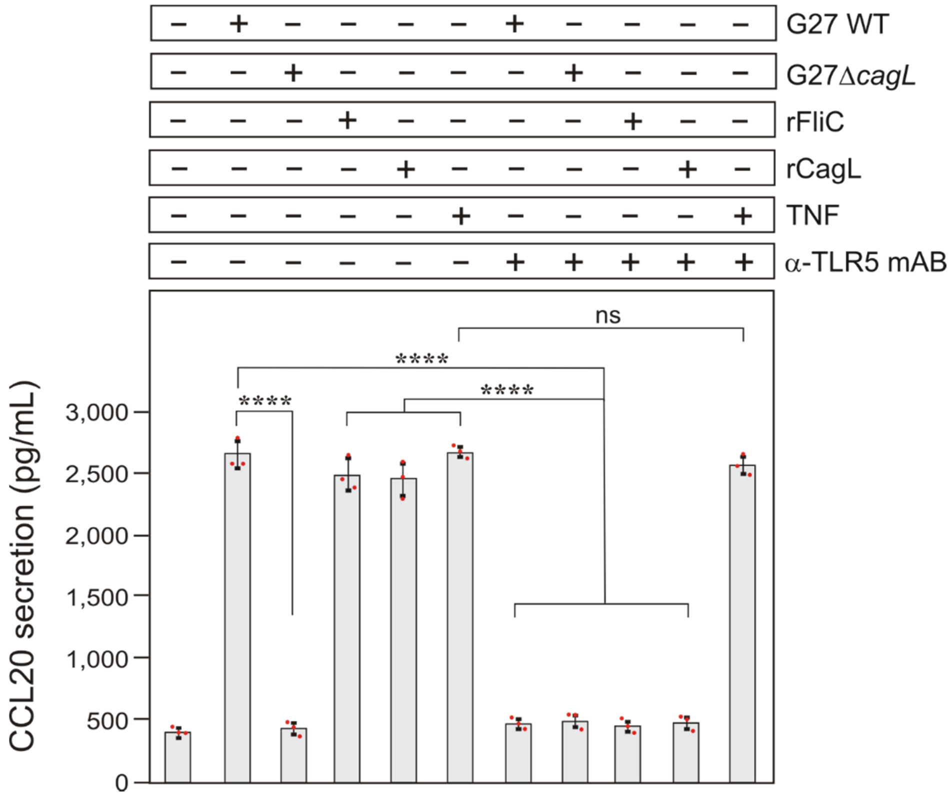

Supplementary Figure 19. CagL-dependent induction of CCL20 cytokine secretion. T84 cells were infected by G27 WT *H. pylori* and Δ*cagL* mutant or treated with recombinant CagL (rCagL, 1 μg/mL) protein for 12 hours. As control, cells were co-incubated with recombinant *Salmonella* FliC (rFliC, 100 ng/mL) or active TNF (10 ng/mL) protein, respectively. As further controls, α-hTLR5 neutralizing antibodies (1 μg/mL) were added in lanes 7-11. *H. pylori*-, rCagL-, rFliC- and TNF-induced CCL20 secretion was measured using standard ELISA. Induced CCL20 secretion was inhibited by the antibody in lanes 7, 9 and 10, confirming that its production is TLR5-dependent. As control for antibody specificity, TNF-induced CCL20 secretion (which signals via TNF receptor) was not inhibited by the antibody (lane 11). All data are representative as means ± SD of three independent experiments; ns: not significant; ****, $p < 0.0001$ (one-way ANOVA). Each red dot represents a single data point. Source data are provided as a Source Data file.

Supplementary Table 1. HEK293-TLR5$^+$ cells infected with *H. pylori*

**Cluster**	**GOTERM_BP_FAT or KEGG_ID**	**Term**	**P-value**	**Genes**
Cluster 1		Enrichment Score: 3.62		
	GO:0071356<br>GO:0034612<br>hsa04060	cellular response to tumor necrosis factor<br>response to tumor necrosis factor<br>Cytokine-cytokine receptor interaction	1.47 E-4<br>1.96 E-4<br>4.79 E-4	CCL2,CCL20, TNFRSF12A, TNFRSF9, TNF
Cluster 2		Enrichment Score: 3.16		
	GO:0032496<br>GO:0002237<br>GO:0009617	response to lipopolysaccharide<br>response to molecule of bacterial origin<br>response to bacterium	1.79 E-5<br>2.23 E-5<br>2.91 E-4	CCL2,CCL20, CXCL1, TNFRSF9, NFKB2, TNF

Supplementary Table 2. HEK293 wild-type cells infected with *H. pylori*

**Cluster**	**GOTERM_BP _FAT or KEGG_ID**	**Term**	**P-value**	Genes
Cluster 1		Enrichment Score: 3.28		
	GO:0006954<br>GO:0034097<br>GO:0002684	inflammatory response<br>response to cytokine<br>positive regulation of immune system process	6.77 E-5<br>1.66 E-4<br>3.34 E-4	CCL2,CXCL1, CXCL3, RELB,TNF
Cluster 2		Enrichment Score: 2.93		
	GO:0048584	positive regulation of response to stimulus	4.55 E-4	CCL2,CXCL1, CXCL3, RELB,NGF,TNF
Cluster 3		Enrichment Score: 2.83		
	GO:0002687<br>GO:0002685<br>hsa04668<br>GO:0032103<br>GO:0032496<br>GO:0002237<br>GO:0050900<br>GO:0030335<br>GO:2000147<br>GO:0051272<br>GO:0040017	positive regulation of leukocyte migration<br>regulation of leukocyte migration<br>TNF signaling pathway<br>positive regulation of response to external stimulus<br>response to lipopolysaccharide<br>response to molecule of bacterial origin<br>leukocyte migration<br>positive regulation of cell migration<br>positive regulation of cell motility<br>positive regulation of cellular component movement<br>positive regulation of locomotion	9.37 E-6<br>2.75 E-5<br>6.79 E-5<br>1.36 E-4<br>1.96 E-4<br>2.24 E-4<br>3.74 E-4<br>4.32 E-4<br>4.78 E-4<br>5.17 E-4<br>5.24 E-4	CCL2,CXCL1, CXCL3,TNF
Cluster 4		Enrichment Score: 2.70		
	GO:0071622<br>GO:0071622<br>GO:0070098<br>GO:0030593<br>GO:1990266<br>GO:0002688<br>GO:0071621<br>GO:0097530	regulation of granulocyte chemotaxis<br>positive regulation of leukocyte<br>chemotaxis<br>chemokine-mediated signaling pathway<br>neutrophil chemotaxis<br>neutrophil migration<br>regulation of leukocyte chemotaxis<br>granulocyte chemotaxis<br>granulocyte migration	1.06 E-4<br>4.95 E-4<br>4.95 E-4<br>5.20 E-4<br>6.50 E-4<br>7.35 E-4<br>7.80 E-4<br>9.54 E-4	CCL2,CXCL1, CXCL3

Supplementary Table 3. Primers used for generating the different CagL mutants

Protein	Primer
$CagL^{L79A}$	*684-fw* *5'-Pho*-TTA TTG AAA GCC AAT TTT GAA GC<br>*684-rv* *5'-Pho*-CGC TGC ATC TCC TCT CAA AGC
$CagL^{L81A}$	*685-fw* *5'-Pho*-GCG GCA TTG AAA GCC AAT TTT GAA G<br>*685-rv* *5'-Pho*-TAA ATC TCC TCT CAA AGC AAT GG
$CagL^{N85A}$	*686-fw* *5'-Pho*-GCC GCT TTT GAA GCG AAT GAG TTA<br>*686-rv* *5'-Pho*-TTT CAA TAA CGC TAA ATC TCC TC
$CagL^{L81A/N85A}$	*684-fw2* *5'-Pho*-GCA TTG AAA GCC GCT TTT GAA GC<br>*684-rv1* *5'-Pho*-CGC TAA ATC TCC TCT CAA AGC

Supplementary Table 4. List of primary and secondary antibodies used in this study

Antibody	Fluorophore	Manufacturer	Catalog No.	Dilution used
CD45	BV650	Biolegend	103151	1/200 (FC)
CD4	BV711	Biolegend	100549	1/200 (FC)
CD4	Fitc	Biolegend	100509	1/200 (FC)
CD4	PerCP	Biolegend	100537	1/200 (FC)
Ly6G	PerCP	Biolegend	127653	1/200 (FC)
TCRβ	PE/Cy7	Biolegend	109221	1/400 (FC)
MHCII	AF700	Biolegend	107621	1/200 (FC)
CD11b	V510	Biolegend	101245	1/200 (FC)
IL-17A	APC	Biolegend	506915	1/100 (FC)
IFNγ	BV421	Biolegend	505829	1/100 (FC)
PY99	---	Santa Cruz	sc-7020	1/1000 (WB)
CagA	---	Austral Biologicals	HPP-5003-9	1/2000 (WB)
His-Tag	---	Qiagen	34670	1/200 (PA)
TLR5	---	Thermo Fisher Scientific	36-3900	1/100 (IHC)
TLR5	---	Santa Cruz	sc-10742	2 µg per sample (IP) 1/500 (WB)
TLR5	---	InvivoGen	maba2-htlr5	10-1000 ng/mL (BA)
TLR4	---	InvivoGen	mabg-htlr4	10-1000 ng/mL (BA)
Flagellin	---	BioGenes	---	1/1000 (WB)
GAPDH	---	BioGenes	---	1/1000 (WB)
CagL	---	BioGenes	---	1/1000 (WB)
CagM	---	BioGenes	---	1/1000 (WB)
CagN	---	BioGenes	---	1/1000 (WB)
Cag3	---	BioGenes	---	1/1000 (WB)
anti-mouse HRP	---	Thermo Fisher Scientific	31446	1/10000 (WB)
anti-rabbit HRP	---	Thermo Fisher Scientific	31462	1/10000 (WB)

Blocking Assay (BA); Flow Cytometry (FC); Immunohistochemistry (IHC); Immunoprecipitation (IP); Peptide Array (PA); Western Blot (WB);

Supplementary References

1. Kwok, T. *et al.* Specific entry of *Helicobacter pylori* into cultured gastric epithelial cells via a zipper-like mechanism. *Infect. Immun*. **70**, 2108–2120 (2002).
2. Yoon, S. I. *et al.* Structural basis of TLR5-flagellin recognition and signaling. *Science* **335**, 859-864 (2012).
