# Supplementary material for: T4SS-dependent TLR5 activation by Helicobacter pylori infection
Source: Nat Commun. 2019 Dec 16;10:5717. doi: 10.1038/s41467-019-13506-6 (PMC6915727; doi:10.1038/s41467-019-13506-6)
Supplement: Supplementary file 2 — Reporting Summary [file 41467_2019_13506_MOESM2_ESM.pdf]

nature research

Corresponding author(s): Steffen Backert

Last updated by author(s): 31.10.2019

Reporting Summary

Nature Research wishes to improve the reproducibility of the work that we publish. This form provides structure for consistency and transparency in reporting. For further information on Nature Research policies, see Authors & Referees and the Editorial Policy Checklist.

Statistics

For all statistical analyses, confirm that the following items are present in the figure legend, table legend, main text, or Methods section.

n/a	Confirmed	
☐	☒	The exact sample size (*n*) for each experimental group/condition, given as a discrete number and unit of measurement
☐	☒	A statement on whether measurements were taken from distinct samples or whether the same sample was measured repeatedly
☐	☒	The statistical test(s) used AND whether they are one- or two-sided<br>*Only common tests should be described solely by name; describe more complex techniques in the Methods section.*
☒	☐	A description of all covariates tested
☐	☒	A description of any assumptions or corrections, such as tests of normality and adjustment for multiple comparisons
☐	☒	A full description of the statistical parameters including central tendency (e.g. means) or other basic estimates (e.g. regression coefficient) AND variation (e.g. standard deviation) or associated estimates of uncertainty (e.g. confidence intervals)
☒	☐	For null hypothesis testing, the test statistic (e.g. *F*, *t*, *r*) with confidence intervals, effect sizes, degrees of freedom and *P* value noted<br>*Give P values as exact values whenever suitable.*
☒	☐	For Bayesian analysis, information on the choice of priors and Markov chain Monte Carlo settings
☒	☐	For hierarchical and complex designs, identification of the appropriate level for tests and full reporting of outcomes
☒	☐	Estimates of effect sizes (e.g. Cohen's *d*, Pearson's *r*), indicating how they were calculated

Our web collection on statistics for biologists contains articles on many of the points above.

Software and code

Policy information about availability of computer code

Data collection	Agilent Feature Extraction Software (FES) for microarray data collection, PDB for crystal structure of zebra fish TLR5 (PDB: 3V47 as template). Multiple protein sequence alignment was performed by applying Clustal Omega program (version 1.2.4).
Data analysis	Modeller 9.16, Swiss-Model38 and RasMol for protein 3D modelling, FlowJo software for Flow cytometry, TIGR MeV 4.9.0 for SAM Analyis and heirarchial clustering, DAVID v6.8 tool for functional annotation of gene expression and Graphpad Prism 8 for statistical analysis were used for the respective functions.

For manuscripts utilizing custom algorithms or software that are central to the research but not yet described in published literature, software must be made available to editors/reviewers. We strongly encourage code deposition in a community repository (e.g. GitHub). See the Nature Research guidelines for submitting code & software for further information.

Data

Policy information about availability of data

All manuscripts must include a data availability statement. This statement should provide the following information, where applicable:

- Accession codes, unique identifiers, or web links for publicly available datasets
- A list of figures that have associated raw data
- A description of any restrictions on data availability

All data generated and analyzed during this study are included in this published Article and its Supplementary Information and Source Data files. The source data underlying Figs. 1, 2a-b, 3b-d, 4e-p, and Supplementary Figs. 1-6, 12, 13, 16 and 19 are provided as a Source Data file.
The microarray data that support the findings of this study have been deposited in the Gene Expression Omnibus (GEO) database with the accession code GSE123623.

Field-specific reporting

Please select the one below that is the best fit for your research. If you are not sure, read the appropriate sections before making your selection.

☒ Life sciences ☐ Behavioural & social sciences ☐ Ecological, evolutionary & environmental sciences

For a reference copy of the document with all sections, see nature.com/documents/nr-reporting-summary-flat.pdf

Life sciences study design

All studies must disclose on these points even when the disclosure is negative.

Sample size	There was no sample size calculation. Adequately and consistently determined samples were used for the measurable differences in this study.
Data exclusions	No data were excluded.
Replication	Replicate experiments as stated in the methods were yielded satisfactory results.
Randomization	There was no randomization, selected samples as stated in the methods were included in the study.
Blinding	No blinding was performed.

Reporting for specific materials, systems and methods

We require information from authors about some types of materials, experimental systems and methods used in many studies. Here, indicate whether each material, system or method listed is relevant to your study. If you are not sure if a list item applies to your research, read the appropriate section before selecting a response.

Materials & experimental systems

n/a	Involved in the study	
☐	☒	Antibodies
☐	☒	Eukaryotic cell lines
☒	☐	Palaeontology
☐	☒	Animals and other organisms
☒	☐	Human research participants
☒	☐	Clinical data

Methods

n/a	Involved in the study	
☒	☐	ChIP-seq
☐	☒	Flow cytometry
☒	☐	MRI-based neuroimaging

Antibodies

Antibodies used	The following commercial available primary antibodies were used in this study: mouse anti-Tetra-His-antibody (Qiagen), anti-mouse CD45 (30-F11), Ly6G (1A8), CD4 (RM4-5), MHCII (M5/114.15.2), CD11b (M1/70) and TCRβ (H57-597) (all from BioLegend), anti-IL-17A (TC11-18H10.1), anti- IFNγ (XMG1.2, Biolegend), polyclonal anti-TLR5 antibodies (Santa Cruz and Thermo Fisher Scientific), monoclonal anti-hTLR5-IgA (InvivoGen), mouse monoclonal anti-phosphotyrosine PY99 (Santa Cruz), rabbit polyclonal anti-CagA (Austral Biologicals) and monoclonal anti-TLR5 (Santa Cruz).<br>Rabbit polyclonal anti-FlaA, anti-CagL, anti-CagM, anti-CagN,anti-Cag3 and human anti-GAPDH antibodies were generated by immunizing rabbits using protein-derived peptides.<br>As secondary antibodies, horseradish peroxidase-conjugated -mouse or anti-rabbit polyvalent goat immunoglobulins were used (Thermo Fisher Scientific).
Validation	No additional validation was performed.

Eukaryotic cell lines

Policy information about cell lines

Cell line source(s)	AGS, HEK293 and T84 cell lines were from ATCC collection and HEK293 cells stably transfected with TLR5 were purchased from Invivogen, France.
Authentication	Cell lines were authenticated externally by commercial vendor and were inspected visually in-house.
Mycoplasma contamination	Cell lines were verified to be free of mycoplasma contamination.

Commonly misidentified lines (See ICLAC register)	Not applicable

Animals and other organisms

Policy information about studies involving animals; ARRIVE guidelines recommended for reporting animal research

Laboratory animals	C57BL/6 WT and tlr5-/- mice
Wild animals	Not used in this study
Field-collected samples	Not applicable
Ethics oversight	All animal experimentation was reviewed and approved by the Zürich Cantonal Veterinary Office (licence ZH24/2013 to A.M.).

Note that full information on the approval of the study protocol must also be provided in the manuscript.

Flow Cytometry

Plots

Confirm that:

☒ The axis labels state the marker and fluorochrome used (e.g. CD4-FITC).

☒ The axis scales are clearly visible. Include numbers along axes only for bottom left plot of group (a 'group' is an analysis of identical markers).

☒ All plots are contour plots with outliers or pseudocolor plots.

☒ A numerical value for number of cells or percentage (with statistics) is provided.

Methodology

Sample preparation	Gastric tissues were cut into pieces, incubated in Hanks' balanced salt solution with 10% FCS and 5 mM EDTA and digested at 37C in RPMI-1640 medium supplemented with 10% fetal bovine serum, 100 U ml−1 penicillin/streptomycin, 15 mM HEPES, 500 U/ml of type IV collagenase (Sigma-Aldrich) and 0.05 mg ml−1 DNase I. Cells were layered onto a 40/80% Percoll gradient, centrifuged, and the interface was recovered and washed in PBS with 0.5% BSA prior to flow cytometry analysis. Mesenteric lymph nodes (MLN) were digested with 500 U/ml of type IV collagenase and mashed through a cell strainer.
Instrument	LSRII Fortessa (BD Biosciences)
Software	Data collection was performed with the BD FACS DIVA software. FloJo software (Tree Star, OR) was used to analyze flow cytometry data.
Cell population abundance	N.A
Gating strategy	All CD45+ leukocytes were pre-gated as live, single, CD45+ cells. CD4+ T cells were then gated as CD4+ TCR a/b+ cells. Neutrophils were gated as CD11b+ MHCII- Ly6G+ cells.

☒ Tick this box to confirm that a figure exemplifying the gating strategy is provided in the Supplementary Information.
